# *Metallicious*: Automated Force-Field Parameterization of Covalently Bound Metals for Supramolecular Structures

**DOI:** 10.1021/acs.jctc.4c00850

**Published:** 2024-10-07

**Authors:** Tomasz K. Piskorz, Bernadette Lee, Shaoqi Zhan, Fernanda Duarte

**Affiliations:** † Department of Chemistry, University of Oxford, Oxford OX1 3QZ, U.K.; ‡ Department of Chemistry—Ångström, Ångströmlaboratoriet Box 523, Uppsala S-751 20, Sweden

## Abstract

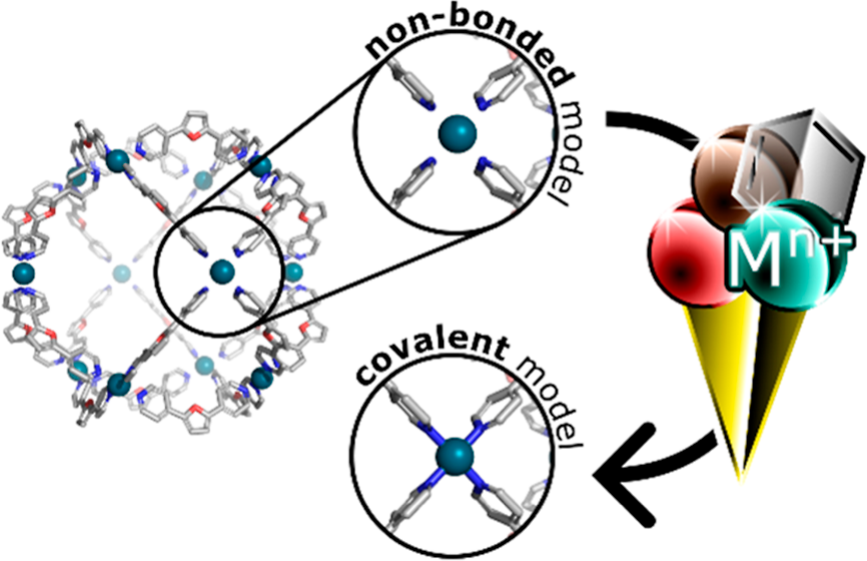

Metal ions play a central, functional, and structural role in many molecular structures, from small catalysts to metal–organic frameworks (MOFs) and proteins. Computational studies of these systems typically employ classical or quantum mechanical approaches or a combination of both. Among classical models, only the covalent metal model reproduces both geometries and charge transfer effects but requires time-consuming parameterization, especially for supramolecular systems containing repetitive units. To streamline this process, we introduce *metallicious*, a Python tool designed for efficient force-field parameterization of supramolecular structures. *Metallicious* has been tested on diverse systems including supramolecular cages, knots, and MOFs. Our benchmarks demonstrate that parameters accurately reproduce the reference properties obtained from quantum calculations and crystal structures. Molecular dynamics simulations of the generated structures consistently yield stable simulations in explicit solvent, in contrast to similar simulations performed with nonbonded and cationic dummy models. Overall, *metallicious* facilitates the atomistic modeling of supramolecular systems, key for understanding their dynamic properties and host–guest interactions. The tool is freely available on GitHub (https://github.com/duartegroup/metallicious).

## 1 Introduction

**Figure 1:**
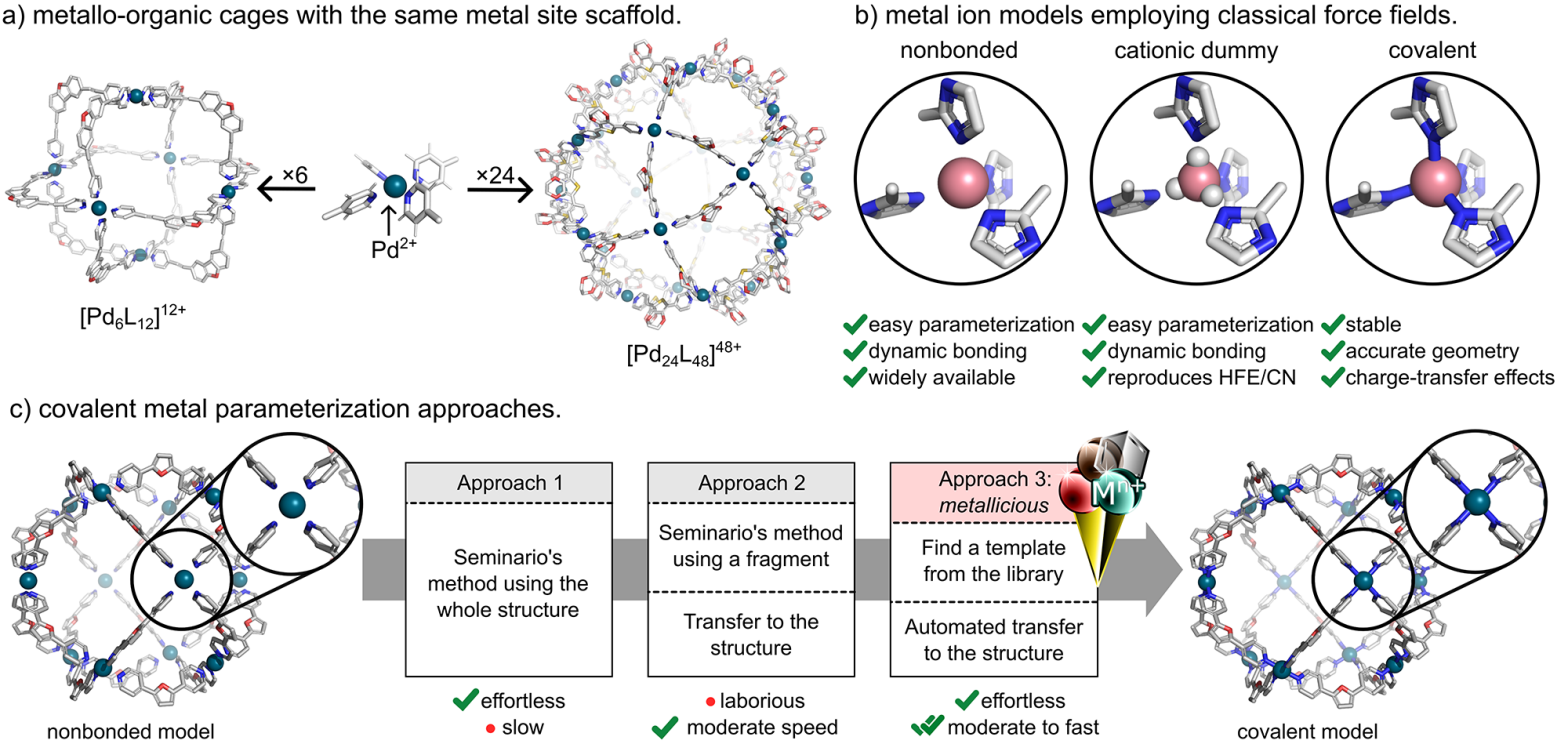
Force-field parameterization of supramolecular systems. (a) Supramolecular metallocages often exhibit repeated metal binding.^91,92^ (b) Approaches for deriving metal force-field parameters include nonbonded, cationic dummy, and covalent models. (c) Methodologies for parameterization of a covalent metal model. *Metallicious* logo appears courtesy of Tomasz K. Piskorz.

Metal ions play significant roles in chemistry, biology, and material science. Approximately one-third of the proteins in the Protein Data Bank contain metals, serving essential structural and catalytic functions.^1^ Metal ions are also key building blocks in supramolecular chemistry, enabling the formation of complex metallo-organic cage structures^2−5^ and metal–organic frameworks (MOFs).^6,7^ Their unique properties stem from their strong directional interactions and coordination patterns, which are unavailable in carbon-based chemistry. Supramolecular structures often feature repeated metal binding sites within their architecture and even across different systems (Figure 1a). For instance, the majority of metal sites in palladium-based supramolecular cages are derivatives of the tetrakis(pyridine)palladium(II) building block.^8^

Various methodologies have been used to model metal-containing systems, including quantum mechanics (QM),^9−12^ molecular mechanics (MM), such as molecular dynamics (MD) and Monte Carlo (MC) simulations,^12,13^ and hybrid QM/MM.^11,12^ These techniques have been applied to model metalloproteins,^14,15^ metallo-organic cages,^16,17^ and MOFs.^18−21^ MM approaches, in particular, offer a significant advantage over QM methods in terms of computational costs.^12^

MM-based approaches for deriving force-field parameters for metal ions include the nonbonded,^22−34^ cationic dummy,^35−44^ and covalent models (Figure 1b).^45−49^ Nonbonded models describe metal centers as van der Waals spheres with integer charge, describing ligand–metal interactions through electrostatic Coulombic and Lennard–Jones (L–J) terms. Li and Merz have reported parameter sets for 56 metals, aiming to reproduce hydration-free energy (HFE), coordination number (CN), and ion-oxygen distances of aqua complexes. However, achieving this often involves identifying parameters that compromise accuracy for these specific observables, since no parameter set can reproduce all of them.^22,23,27−30^ Zhang sampled a larger L−J parameter space to identify parameters capable of reproducing HFE and CN for a set of 47 ions.^31−34^

The dummy model, originally developed by Åqvist and Warshel,^24,50^ places cationic dummy particles bonded to metal in a predefined coordination geometry. These particles exclusively interact with ligands via electrostatic interactions. This model provides an improvement over nonbonded models by simultaneously reproducing HFE and CN. However, its predefined configuration reduces flexibility, and only a limited number of metal ions have been parameterized.^35−42,44^ While both nonbonded and cationic dummy models can describe bond-forming and -breaking processes,^42,43,51,52^ they often exhibit unexpected behavior when transferred to different systems^35,53^ and fail to account for charge transfer.^43,54,55^

The covalent bond model explicitly describes bonds, angles, torsions, and van der Waals and Coulombic interactions between coordinating ligands and metal ions, allowing it to account for charge transfer effects but unable to account for ligand exchange. Parameterizing the different terms is time-consuming, often relying on equilibrium-bonded parameters and force constants obtained from QM calculations. The two most common approaches for obtaining bond force constants involve calculating the Hessian matrix^49,56−59^ or employing energy-fitting or/and force-matching techniques.^60−64^ Among Hessian-based methods, the Seminario method is the most widely used. It involves the projection of the Hessian matrix elements onto relevant bonded parameters using the harmonic approximation (Figure 1c; approach 1).^56^ In this approach, dihedral parameters involving metal ions are omitted as they usually have minimal impact on the structure,^65−68^ although exceptions including these terms exist.^69^ This method has been successfully applied to model small metallo-organic molecules,^70−72^ metalloproteins,^67,73^ MOFs,^74−76^ and supramolecular cages.^77−79^ Cole and co-workers have improved the Seminario method by addressing the problem of double-counting bending interactions.^57^ Alternatively, the energy- and force-matching techniques fit energies and forces to reproduce the reference data.^64,80,81^ These methods have also been successfully applied to metallo-organic molecules^82^ and cages.^83^ Hu and Ryde have compared these two approaches for Zn^2+^ complexes, finding that while energy- and force-matching techniques more accurately reproduced QM-optimized geometry of the complexes, they were also more laborious, requiring significant human intervention.^84^ The L−J parameters are typically taken from nonbonded model parameters or the Universal Force Field, which covers 126 elements,^85^ while partial charges are obtained using the restrained electrostatic potential (RESP) method,^86^ available in various software including AmberTools (via Gaussian, GAMESS-US),^87^ R.E.D. server (Gaussian, GAMESS-US, Firefly),^88^ psi4/resp (psi4),^89^ and psiRESP (psi4).^90^

The Seminario method and RESP calculations are computationally costly, restricting their use to small systems. For larger structures, such as metalloproteins, a cluster representing the metal binding site is often used for parameterization (Figure 1c; approach 2). These parameters are then transferred to the larger system, usually involving significant manual intervention. To speed up this process, Li and Merz developed MCPB.py,^93^ offering a semiautomatic approach to derive bonded parameters and charges. MCPB.py is compatible with the AMBER force field, and while it is suitable for proteins with a few distinct metal sites, its use becomes laborious for supramolecular structures, as they often consist of multiple identical metal sites. Due to differences in atom order, each site would require separate QM calculations, resulting in a time-consuming procedure. Ideally, an approach similar to those available for protein parameterization (e.g., *pdb*2gmx in GROMACS and *LeAP* in AMBER), where residue parameters are tabulated and automatically assigned, tailored to metal sites and neighboring residues would significantly streamline the modeling of supramolecular structures containing repeating metal site units (Figure 1a).

It is important to note that more advanced force-field potentials for specific metal ions have been developed, for example, using a polarizable force field, such as the classical Drude oscillator model,^94^ or more advanced models, including atomic multipole moments^95^ or electrostatic penetration.^96^ Another promising avenue has been the development of a generic force field by Grimme et al.,^97^ which includes parameters for most metals and has been used for gas-phase optimization of MOFs and metallo-organic cages.^21^ While such approaches are usually more accurate than standard force fields, they remain computationally too expensive for routine all-atoms condensed-phase MD simulations.

In this work, we introduce *metallicious* (Figure 1c; approach 3), a tool designed to streamline the parameterization of metal centers using the covalent metal model based on a *template library*. It enables the parameterization of new templates using Seminario’s method and RESP charges. As a result, *metallicious* has increased efficiency and applicability to systems with repetitive metal units, including supramolecular cages, MOFs, and knots.

## 2 Methodology

In the following paragraphs, we explain the structure and functionalities of *metallicious*.

### 2.1 Code Structure

**Figure 2:**
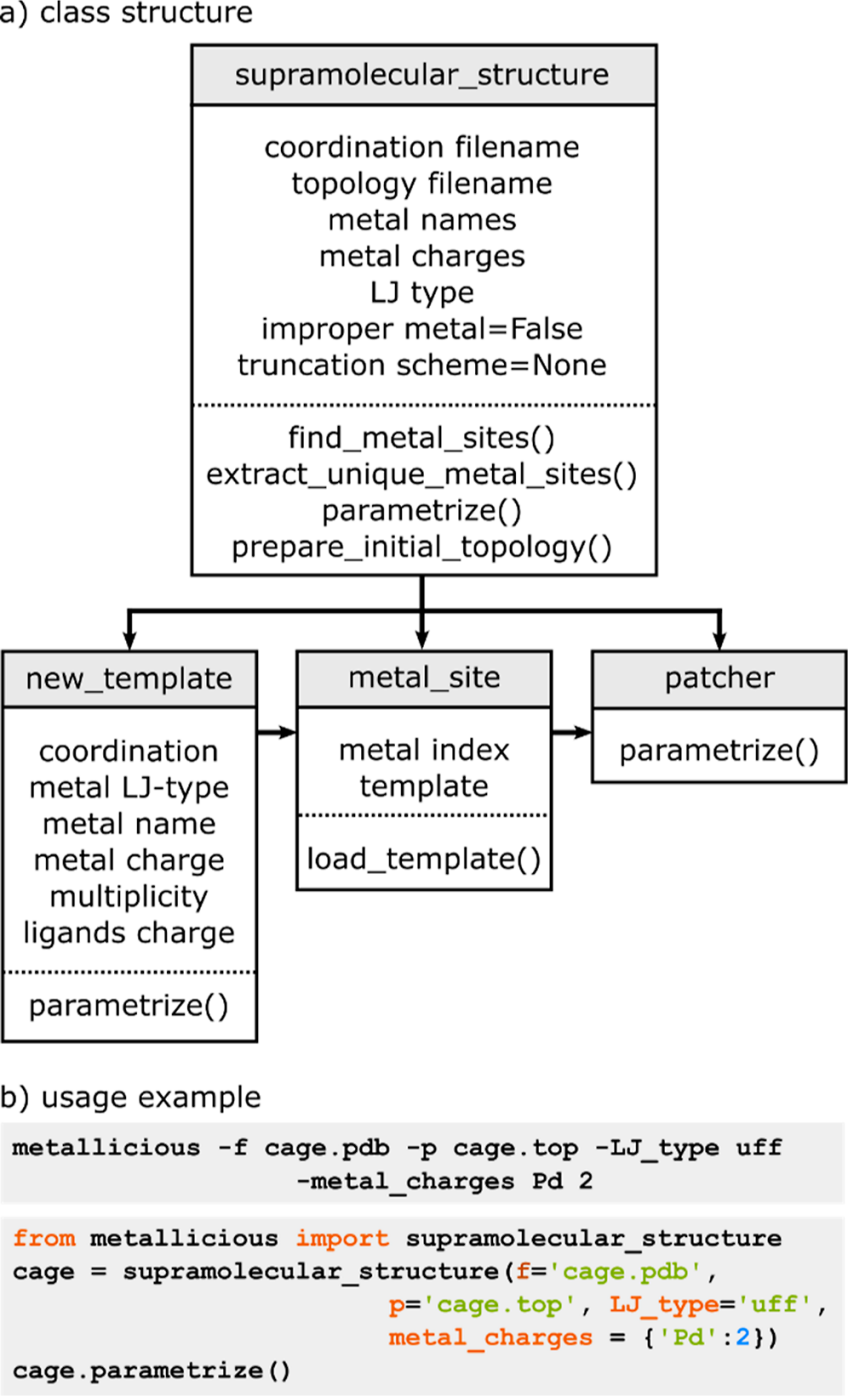
*Metallicious* code structure. (a) Class structure of *metallicious*, including its four main objects and their relationship. (b) *Metallicious* can be executed via either a command line or Python script. Input requirements include coordinate (.pdb), topology (.top), L−J type, and identity and charge of the metal ion.

*Metallicious* is written in object-oriented Python and uses scientific programming modules, including NumPy,^98^ MDAnalysis,^99^ RDKit,^100^ ParmEd,^101^ and NetworkX^102^ for core functionalities. Optionally, the parameterization of new templates requires the QM engine ORCA,^103,104^ autodE (used as an interface for the QM engine),^105^ and psiRESP,^90^ and parameterization of the organic molecules requires AmberTools.^106^ The code is built around four key objects (*supramolecular_structure, new_template, metal_site, and patcher*). The relationship between these objects is outlined in Figure 2a.

The *supramolecular_structure* class serves as the central element within the module, holding information about the overall code structure. It encompasses the subclasses *new_template*, *metal_site*, and *patcher*, as well as their associated coordination and topology files. Core functionalities include extraction of metal sites from the *coordinate file*, identification of a suitable template from the database, and execution of template parameterization. For each metal site identified in the input, a *metal_site* class is generated, storing the metal index and the corresponding template. Preparameterized templates are stored in a dedicated subdirectory within *metallicious* (in GROMACS topology format). In cases where the template is absent, a *new_template* class is created, containing all information necessary to parameterize the new metal site (truncated coordinate file, metal name, charge, multiplicity, L−J parameters, and ligand charges). Lastly, the *patcher* class combines all of the *metal_site* classes with the input structure and topology to produce the output files.

### 2.2 Code Usage

*Metallicious* can be executed either via a command line or imported as a Python script (Figure 2b). The tool requires the following input information:

(a) *Coordinate file (f).* Accepted formats include .xyz, .pdb, .gro, and others supported by MDAnalysis. The coordination file might be taken directly from the Cambridge Crystallographic Data Centre (CCDC) (after removing solvent and additives), generated using our tool for automated metallocage construction, *cgbind*,^107^ or Avogadro and optimized with the Universal Force Field.^108^
(b) *Topology file (p)* or specify *prepare_initial_topology* argument. Topology file containing force-field parameters for the input coordination file. Accepted formats include .top, .itp, .prmtop, and other files supported by ParmEd.^101^ Note that the L−J parameters and charge of metal atoms present in the topology files are not used, only their indices. Such parameters can be obtained from tools such as AmberTools,^106^ ATB,^70^ or CHARMM-GUI.^109^ Instead of providing a topology file, the user might request them by specifying the *prepare_initial_topology* argument, which interfaces with the antechamber to obtain general AMBER force field parameters for the ligand.^106^
(c) *LJ type (LJ_type)*. Specify the name of the library used to extract the metal L−J parameters. Options include Merz, Zhang, or UFF libraries.
(d) *Metal name(s) and charge(s) (metal_and_charge)* and *optionally multiplicity.* This information is used to identify the metal ions in the input structure and the template to be used. Multiplicity is required only for the parameterization of new templates.

For new templates, users can also include parameterization of an improper dihedral formed by metal and bound aromatic molecules, which is turned off by default (Section S4.1). This can be done by specifying the *improper_metal* option. Additionally, several truncation schemes of templates are available to simplify parameterization, which are also turned off by default. A full description of the input is provided in Table S1.

Upon completion of the parameterization, two output files are generated: a coordination file with reordered atoms (default: out.pdb) and a topology file saved in GROMACS format (default: out.top), although other formats are supported via ParmEd. The topology file contains modified bonds, angles, dihedrals, and partial charges of the metal and its surrounding atoms and modified L−J parameters of metals.

### 2.3 Limitations

The code currently supports organometallic structures with metals separated by at least two nonmetal atoms; metal clusters are not currently supported.

### 2.4 Code Functionality

**Figure 3:**
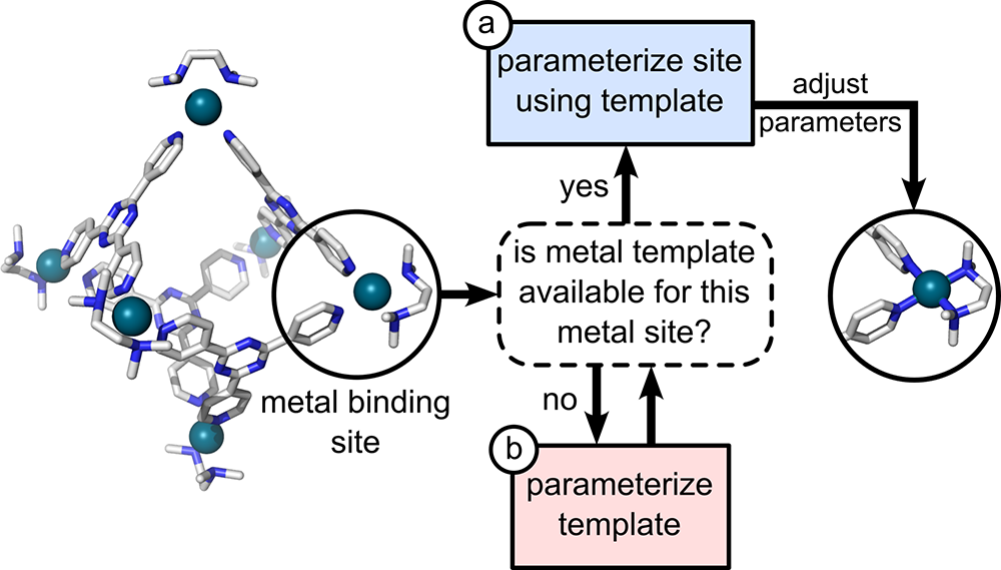
Overview of metal parameterization in *metallicious*, illustrating the communication between the two primary program subroutines: parameterization site using a template and (new) template parameterization.

As illustrated in Figure 3, *metallicious* iterates through each metal center in the input structure, executing one of the following actions:

(a) Parameterization using a template library. Selects a suitable template from a repository of predefined templates and adjusts the input topology accordingly.
(b) Parameterization of a new template. If a suitable template is unavailable, it performs template parameterization.

#### 2.4.1 Parameterization Using a Template Library

**Figure 4:**
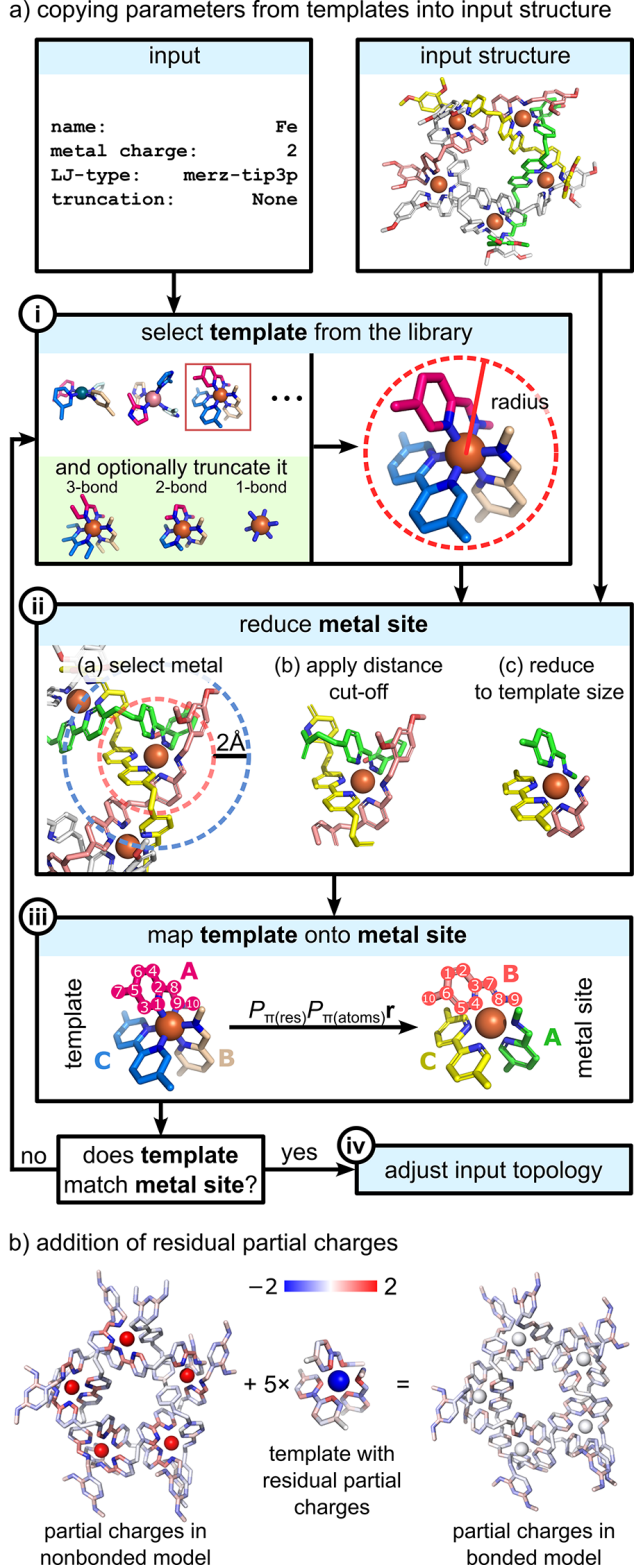
Parameterization workflow using templates: (a) Copying parameters from a template into the input coordination and topology files. *P*_π(res)_ and *P*_π(atoms)_ denote permutation matrix over residues and residues’ atoms, respectively, and **r** denotes atom positions. (b) Partial charges accounting for charge transfer are obtained by summing partial charges from the nonbonded model and template’s residual partial charges.

The most efficient approach is to use readily available templates. This subroutine is always performed for each site and can be summarized in the following key steps (Figure 4a):

##### 2.4.1.1 Template Selection and (Optionally) Truncation

This search aims to identify file names that match the user-specified metal name, charge, and L−J type (Figure 4a.i). The first matching template is used for the next step. If unavailable, users can truncate the system and perform the search again or parameterize a new template. Truncation involves applying a distance cutoff based on the user-defined truncation_scheme variable (default: none for which the whole template structure is considered). Depending on the scheme, atoms more than 1, 2, or 3 bonds away from the metal center are removed, and their charges are evenly redistributed among the remaining atoms. The influence of different truncation schemes on the final parameters is discussed in Section 3.3.

##### 2.4.1.2 Reduction of the Metal Site

The original metal site is aligned with a selected template, chosen after matching the name, charge, and L−J type through an iterative process. A distance cutoff equal to the template’s radius plus 2 Å is used [Figure 4a.ii, blue dashed line]. The template’s radius is defined as the distance between the metal and its furthest atom [Figure 4a.ii, red dashed line]. Matching ligands in both structures are identified by constructing their molecular graphs and locating combinations where the template’s linker graphs are subgraphs of the metal site’s graphs. The metal site’s linkers are then pruned to match template graphs, resulting in identical structures that only differ in the order of the atoms.

##### 2.4.1.3 Mapping of the Template onto the Metal Site

**Figure 5:**
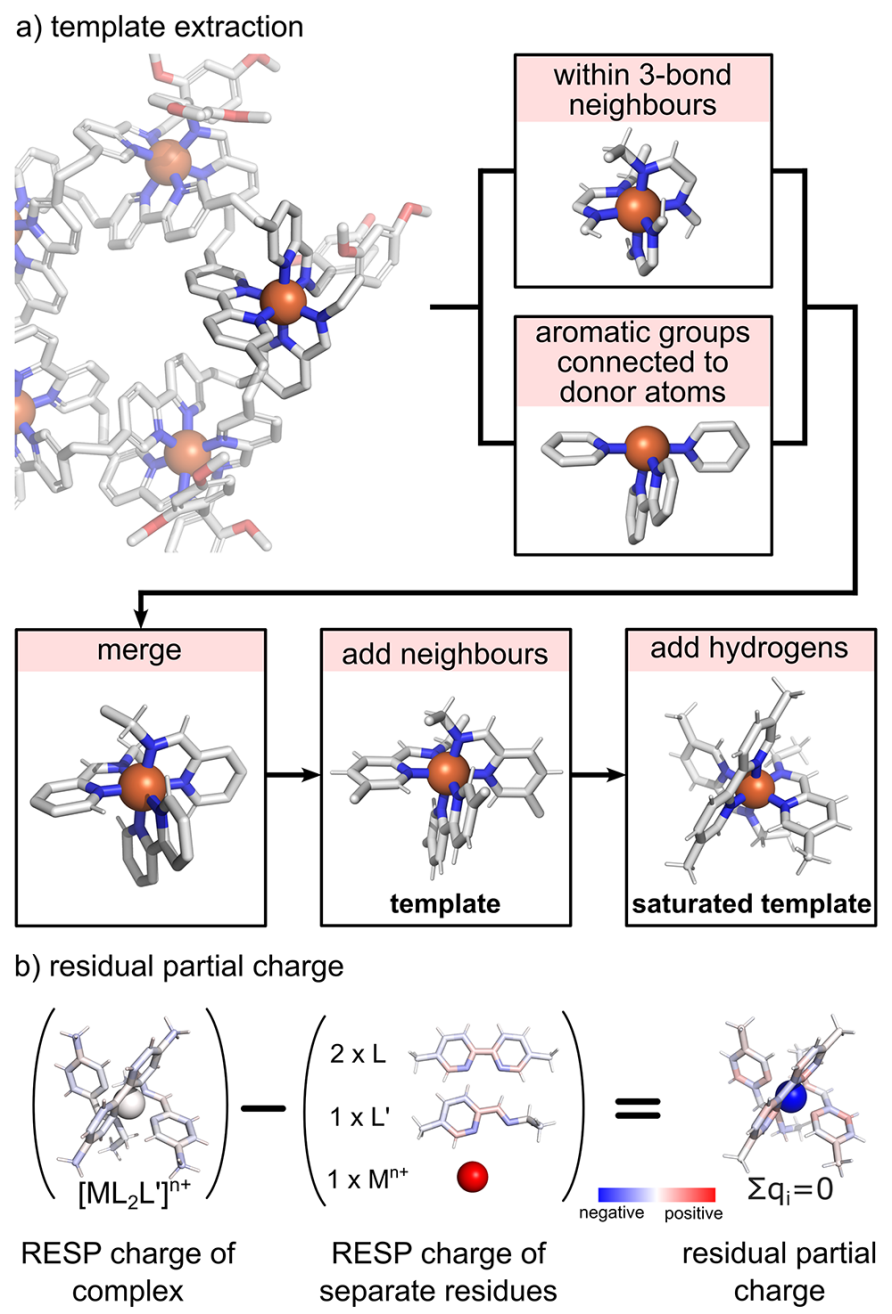
New template parameterization. (a) Extracting the metal template from the supramolecular complex. (b) Calculating the residual partial charge of the template.

Achieving the correct atom order involves an exhaustive exploration of all possible permutations of ligand and atom numbering, the most time-consuming part of the process (Figure 4a.iii). For efficiency, the process is divided into two stages. First, the order of all non-hydrogen atoms is determined by exhaustively exploring permutations of the pruned metal site and selecting the one with the lowest root-mean-square displacement (RMSD) with the template with reordered atoms (Figure S1). Subsequently, hydrogen atoms are reconstructed by finding an isomorphism between the template’s and the metal site’s linker graphs, constraining the order of heavy atoms. Successful mapping is defined as RMSD <2 Å, although this criterion can be modified. If RMSD >2 Å, the next template from the library is evaluated; if unavailable, the procedure for parameterizing a new template is followed (Figure 5).

##### 2.4.1.4 Adjusting Input Topology

If the steps above are successful, the structure’s topology is updated by substituting its bonded parameters with those from the reordered template (Figure 4a.iv). This topology is further updated by adding residual partial charges from the template, which account for charge transfer effects (Figure 4b; for comparison with RESP charges, see Figure S2). These partial charges are obtained from the differences in charges between the complex and separate species (vide infra, Figure 5b). Since the sum of residual charges of the template is zero, the result of its addition to the new topology only results in the redistribution of partial charges.

#### 2.4.2 Parameterization of a New Template

When no template is found in the library, *metallicious* performs parameterization using the following steps:

##### 2.4.2.1 Extraction of a New Template

To extract the new template, *metallicious* iterates through metal atoms in the input file (Figure 5a). It selects atoms that are either (a) within a distance of 3 bonds from the metal or (b) part of a connected aromatic group(s) coordinated with the metal. The selected atoms from both categories are combined to form the template’s backbone. Atoms that are not part of the template’s backbone but directly connected to it by one bond distance are added to the structure, resulting in the *template*, which is the main structure for parameterization using the template library. Hydrogen atoms are added to atoms with unfulfilled valence, saturating the template, which is required for QM calculations (*saturated template*).

##### 2.4.2.2 Parameterization of Bonded Parameters

Bonds and angles are parameterized using Seminario’s method, adapted from Cole and co-workers.^57^ This uses the Hessian matrix computed via ORCA/autodE for the *saturated template*. Equivalent bonds and angles are symmetrized by comparing molecular graphs of metal–ligand pairs (Figure S4). Similar to the work of others, proper dihedrals are not parameterized.^65−68^ Parameters for improper dihedral are obtained by performing a 1D scan involving the metal ion and the donor atoms. We found that including improper parameters improves the geometry of some metal sites (vide infra, Section 3.2).

##### 2.4.2.3 Residual Partial Charge

Partial charges are calculated using the RESP method.^86^ During this process, the electrostatic potential (ESP) is computed at the D3BJ-PBE0/def2-SVP level of theory using ORCA for the individual ligands and the saturated template separately. This methodology was selected based on the wide availability of the basis set for most elements of the periodic table and the robustness and efficiency of the hybrid PBE0 functional.^110−113^ Moreover, this level of theory was found to provide similar results to the popularly used B3LYP/6-31+G*. (Figure S2). The total charge of the saturated template, required for initiating QM calculations, is determined by summing up the user-specified metal charge and RDKIT-generated ligand charges. Subsequently, the partial charges are computed using psiRESP. During this process, charges of linking atoms are constrained to zero. From these calculations, a residual partial charge is computed by subtracting the partial charges of the *saturated template* from the partial charges of individual ligands and the metal (Figure 5b). Lastly, the obtained charges are symmetrized for identical ligands, determined using the isomorphism of their molecular graphs.

##### 2.4.2.4 Map Parameters onto the Template

The additional atoms added to form the *saturated template* are removed, resulting in the final *template*, which is added to the template library.

## 3 Results

To assess the capabilities of *metallicious*, we considered 11 supramolecular systems whose structures were obtained from the Cambridge Crystallographic Data Centre (CCDC), including seven cages ([Pd_2_L_4_]^4+^,^114,115^ [Ga_4_L_6_]^12–^,^116^ [Fe_4_L_6_]^4–^,^117^ [Pd_6_L_4_]^12+^,^118^ [Co_8_L_12_]^16+^,^119^ [Pd_6_Ru_8_L_24_]^28+^,^120^ and [Pd_48_L_96_]^96+^),^121^ two knots ([Fe_5_L_5_]^10+^,^122^ [Zn_3_L_3_]^6+^),^123^ and two MOFs (ZIF-8^124^ and ZIF-67;^125^Table S2). Among these systems, [Pd_2_L_4_]^4+^,^126^ [Ga_4_L_6_]^12–^,^77,127^ [Pd_6_L_4_]^12+^,^78^ ZIF-8,^74^ and ZIF-67^75^ have been previously modeled using MD simulations. The others were selected due to their technical challenges for automation. For example, [Co_8_L_12_]^16+^ contains eight metal sites, four of which are stereoisomers, differing only by orientation of the ligands around the metal; [Pd_6_L_4_]^12+^ comprises two different ligands, pyridine and diamine; [Pd_6_Ru_8_L_24_]^28+^ requires routines to handle two distinct metals and ligands. Finally, [Pd_48_L_96_]^96+^ was chosen, as it is the largest synthesized supramolecular cage to date.

**Figure 6:**
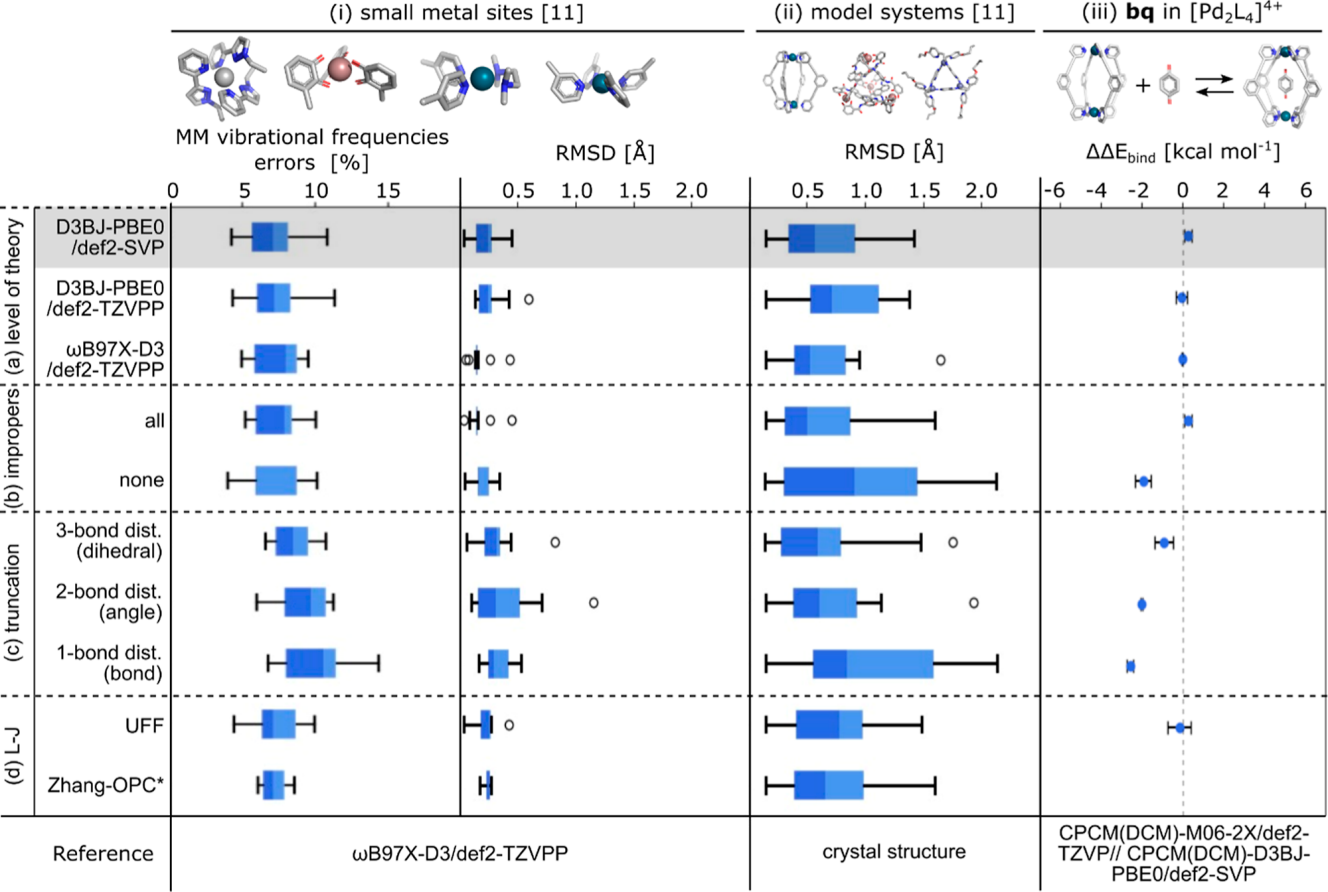
Benchmark of *metallicious* considering (i) 11 small metal sites, including a comparison of MM- and QM-computed vibrational frequencies and RMSD values between MM- and QM-optimized structures, (ii) 11 representative supramolecular systems, including comparison between MM-optimized and crystal structures, and (iii) benzoquinone (**bq**)-[Pd_2_L_4_]^4+^ complex, comparing MM and QM binding energies. The benchmark considered the influence of (a) different levels of theory on parameterization, (b) inclusion of improper parameters, (c) truncation scheme, and (d) type of L−J parameters (Zhang-OPC parameters are available only for nonpalladium systems). Except where specified by (a–d), the template was parameterized at the D3BJ-PBE0/def2-SVP level of theory; “none” for the truncation scheme; Merz-OPC L–J parameters and improper parameters included only for systems with square-planar complexes (gray shaded area). The dark and light blue colors represent lower and upper interquartile ranges, respectively.

The metric used to assess the quality of the parameters includes comparing the MM- and QM-computed vibrational frequencies and optimized structures for 11 small metal sites (Figure 6i; see Figure S5 for all structures), RMSD values between MM-optimized and crystal structures of 11 representative supramolecular systems (Figure 6ii), and MM and QM binding energies for the benzoquinone (**bq**)-[Pd_2_L_4_]^4+^ complex (Table S3), with the MM value obtained from five different starting structures (Figure 6iii).

By default, the templates were parameterized (Section 2.4.2) at the D3BJ-PBE0/def2-SVP level of theory without truncation using the Merz-OPC L–J parameters; improper parameters were included only for systems with square-planar complexes. Using the default parameters shows good performance across the different metrics evaluated (Figure 6). For example, when comparing vibrational frequencies for the small metal sites, the mean percentage error is only 6.7%, similar to the 6.4% obtained by Cole and co-workers for 70 small molecules using the modified Seminario method.^57^ The MM-optimized small metal sites also result in structures similar to those optimized by QM (RMSD <0.5 Å). Similarly, good agreement is obtained when comparing the MM-optimized and crystal structures of supramolecular systems (RMSD <1.5 Å). Lastly, the binding energy of (**bq**)-[Pd_2_L_4_]^4+^ shows excellent agreement with QM-computed energies (<0.5 kcal mol^–1^). These results indicate that the parameterization obtained through *metallicious* is robust enough to obtain not only structural energetics but also host–guest energetics.

### 3.1 Influence of the Level of Theory on Quality of Parameters

We first assessed the impact of different functionals (D3BJ-PBE0 and ωB97X-D3) and basis sets (def2-SVP and def2-TZVPP) on the quality of the parameters obtained from the Seminario method (Figure 6a). The results obtained for the different benchmarks were similar regardless of the functional and basis set used. Surprisingly, despite the reference values being derived from calculations at the ωB97X-D3/def2-TZVPP level of theory, the parameters obtained at this level of theory were not better than those obtained at the D3BJ-PBE0/def2-SVP level of theory. This observation suggests that the loss of accuracy during parameterization originates from the inadequate depiction of interactions by conventional force fields. For example, harmonic bonds might poorly approximate metal–ligand interactions.^128^ Since D3BJ-PBE0/def2-SVP is computationally efficient, it is used as a default methodology to parameterize templates in *metallicious*.

### 3.2 Importance of Improper Dihedral Parameters Involving Metals

**Figure 7:**
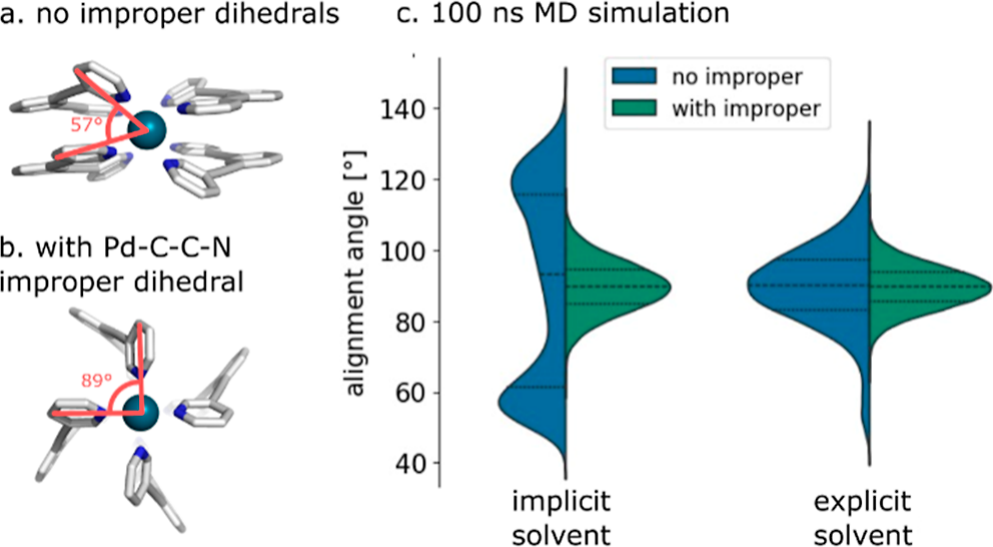
Importance of improper dihedral parameters involving metals. Energy-minimized structures using L-BFGS with GBSA implicit solvent (a) with and (b) without the improper dihedral. (c) Histogram of the angle between two ligands from 100 ns MD simulations of the [Pd_2_L_4_]^4+^ cage with implicit and explicit DMSO solvent.

Proper and improper dihedral parameters involving metal centers are rarely considered as they are generally thought to have little impact on geometry.^65−68^ However, exceptions have been reported for square-planar Ni^2+^ complexes, where including the out-of-plane bending term was necessary to reproduce crystal structures.^69^ Similarly, during MD simulations of the [Pd_2_L_4_]^4+^ cage in implicit solvent, we observed deviation from the expected square-planar geometry when improper dihedrals centered on the donor atom were not included (Figure 7a). Including improper dihedrals centered on the donor atom yielded structures similar to those obtained from QM in an implicit solvent (Figure 7b). Interestingly, these parameters can be omitted in explicit solvent, likely due to the dominant effect of solvent–solute interactions over solute–solute stacking interactions. For example, during 100 ns MD simulation in explicit dimethyl sulfoxide (DMSO), when no metal-involved improper dihedrals are included, less than 10% of the configurations adopted a staggered configuration compared to 50% in implicit solvent (Figure 7c).

Optimization of all other structures with and without improper dihedrals in implicit solvent demonstrated that this issue is specific to systems featuring a square-planar configuration (Figures 6b and S6). Otherwise, including improper parameters has no impact on the geometries. For this reason, and considering the computational cost associated with obtaining these parameters, their parameterization is disabled by default in *metallicious*, except for Rh^+^, Ir^+^, Pd^2+^, Pt^2+^, and Au^3+^, which often have square-planar configurations.

### 3.3 Truncation Schemes

**Figure 8:**
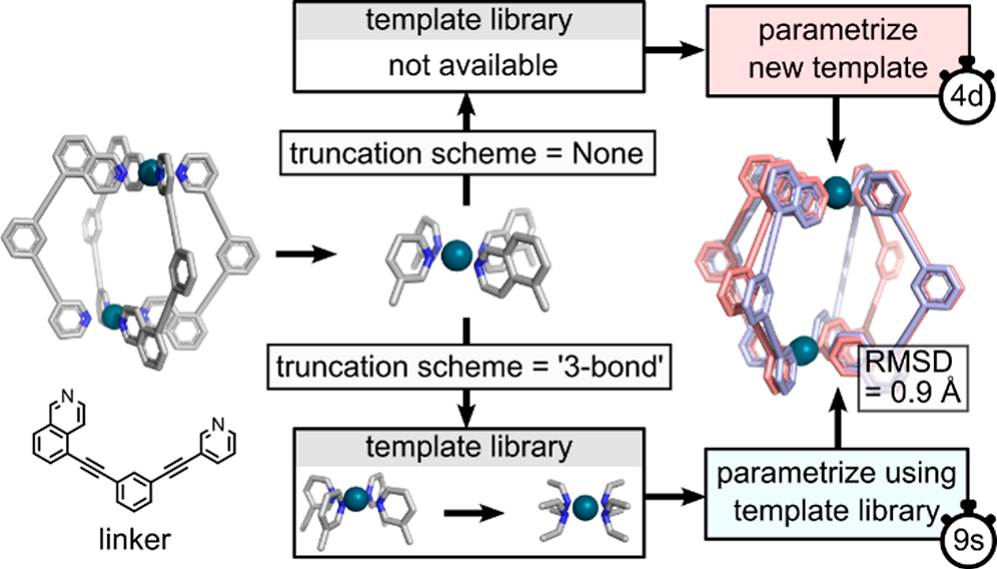
Truncation scheme. This scheme speeds up calculations by replacing missing templates with substructures from related templates. Timing is shown for a single CPU@2.5 GHz.

Deriving new templates in *metallicious* is fully automated but computationally intensive, especially for systems with unique metal sites like the asymmetric cage reported by Lewis et al. (Figure 8)^129^

To expedite this process, we created truncation schemes that reuse existing templates. The degree of truncation depends on the selected scheme: none, 3, 2, and 1 bond distance from the metal center. For the example mentioned above, which currently lacks a template, *metallicious* parameterizes a new template in around ∼100 CPUhs compared to only a few seconds when it uses a template library by applying a 3 bond distance truncation scheme to an existing template. While truncation schemes expand the utility of the existing template library, they sacrifice accuracy and therefore must be used with caution. The benchmarks reveal that the accuracy loss is proportional to the extent of truncation (Figure 6c). It is therefore recommended, when possible, to start parameterization with the least truncated template.

### 3.4 L–J Parameters

The effect of the use of different L–J parameters (Merz-OPC, UFF, and Zhang-OPC) on the evaluated metrics was found to be minimal (Figure 6d). This is likely due to the dominance of the bonded parameters.

### 3.5 Application of *Metallicious*

**Figure 9:**
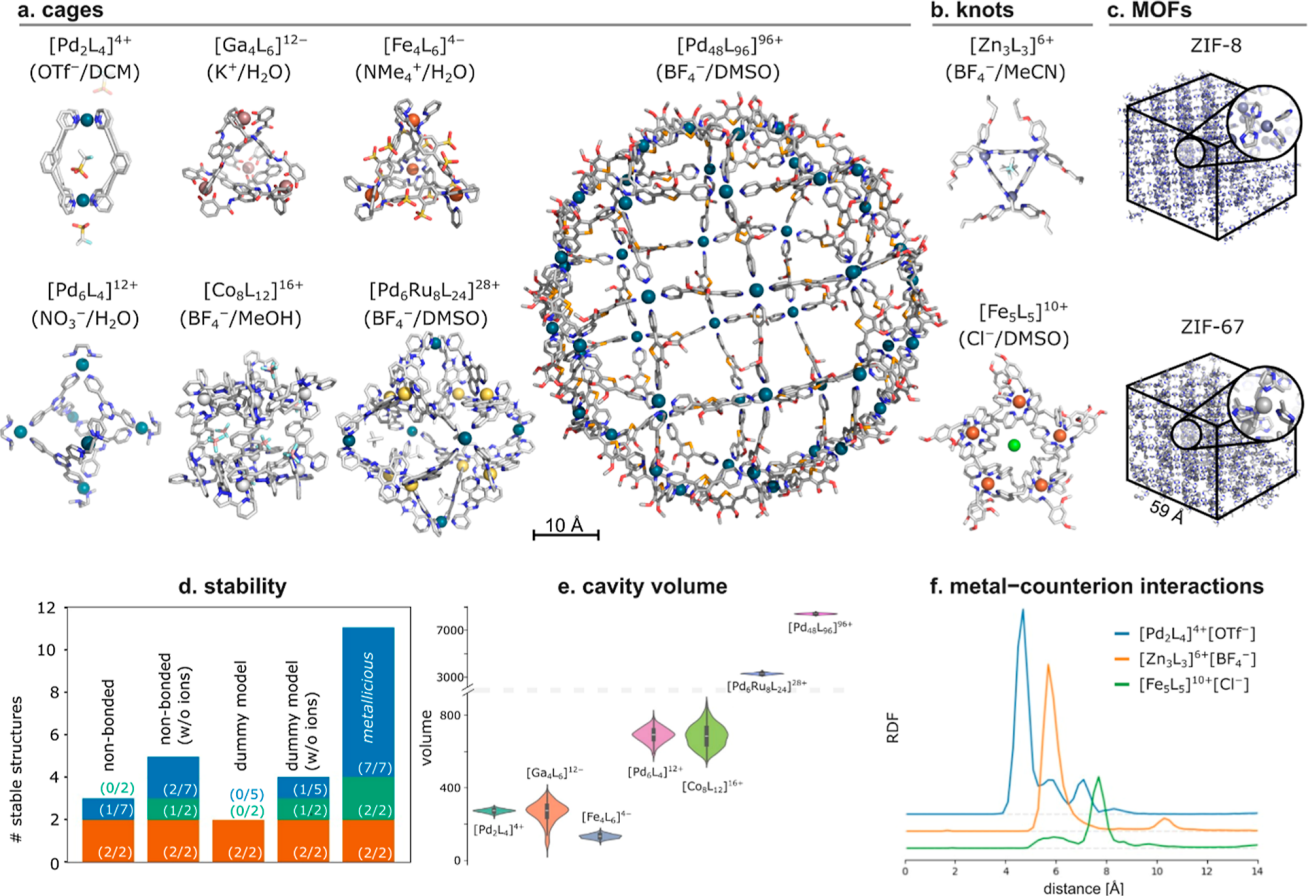
Analysis from 100 ns MD simulations for systems parameterized by *metallicious*. Final snapshots for (a) cages, (b) knots, and (c) MOFs (solvent and counterions not shown for clarity, except for [Pd_2_L_4_]^4+^, [Zn_3_L_3_]^6+^, and [Fe_5_L_5_]^10+^). (d) Histogram of stable structures after simulation (blue—metallo-organic cages, green—supramolecular knots, and orange—MOFs). (e) Distribution of cavity volumes calculated using *C3*.^132^ (f) RDFs calculated for metal centers and counterions (see Figure S20 for further details). DCM = dichloromethane; DMSO = dimethyl sulfoxide; MeOH = methanol; MeCN = acetonitrile; OTf^–^ = triflate; NMe_4_^+^ = tetramethylammonium; NO_3_^–^ = nitrate; BF_4_^–^ = tetrafluoroborate.

MD simulations provide access to the dynamics properties of supramolecular systems that cannot be obtained from crystal or QM-optimized structures. *Metallicious* simplifies the setup of such simulations, facilitating the analysis of dynamic properties and their implications for host–guest interactions. To illustrate this, we parameterized and performed each MD simulation for 100 ns of the 11 supramolecular systems described above (Figure 9a–c). Except for ZIF-8 and ZIF-67, which lack counterions and solvent molecules in their crystals, simulations were conducted in explicit solvent and included counterions, which often strongly interact with metals. We compared the results with simulations using the nonbonded^29,85^ and dummy metal models,^35,42,44^ evaluating stability of the simulations through changes in the metal coordination sphere.

As anticipated, structures using covalent metal models remained stable over the simulation time (Figure 9d; see Table S4 and Figures S7–S17 for RMSD, coordination sphere analysis, and snapshots of individual trajectories), with a median RMSD = 1.1 Å relative to the crystal structure. In contrast, most of the simulations utilizing nonbonded and dummy models resulted in disassembly. Only ZIF-8 and ZIF-67 MOFs produced stable simulations with these models, possibly due to the absence of competing interactions with counterions and solvent. Removing counterions in the other systems resulted in a marginal improvement in stability. While the stability of the covalent model is expected as dissociation is not allowed, the poor performance of other models for almost all systems was surprising. This is likely the result of parameters being overfitted to reproduce aqueous complexes rather than interactions with other heteroatoms containing molecules, leading to imbalanced metal–ligand and metal–counterion interactions.

Analyzing the flexibility of the cages provides a more realistic picture of how much a cage cavity changes and adapts to different guests. Among the simulated cages, the [Ga_4_L_6_]^12–^ cage shows the largest relative change in volume, from <1 Å^3^ (no grid point can be placed inside) to 267 Å^3^ (Figure 9e; Figure S19). This flexibility is due to the rotatable central naphthalene linker. Indeed, the [Ga_4_L_6_]^12–^ cage has been found to bind a broad range of substrates, including quaternary ammonium cations with volumes ranging from 80 to 160 Å^3^.^127,130^

Furthermore, an analysis of the interactions between the supramolecular systems and counterions can be done by computing the corresponding radial distribution functions (RDFs; Figure S20). For example, the [Pd_2_L_4_]^4+^ cage reveals a prominent peak at 4.7 Å, indicative of a conserved interaction between the cage and the triflate counterion (Figure 9f). Notably, Lusby and co-workers showed that this counterion exhibits strong binding affinity to the [Pd_2_L_4_]^4+^ cavity.^115^ Similar results were observed for the [Zn_3_L_3_]^6+^ and [Fe_5_L_5_]^10+^ knots, indicating a strong binding interaction at 5.7 and 7.7 Å, respectively. Indeed, Leigh and co-workers showed that the [Fe_5_L_5_]^10+^ knot can extract chloride traces from solvent and glassware.^131^

## 4 Conclusions

We developed an automated tool called *metallicious* for parameterizing covalent metal models in supramolecular structures. Our method leverages the repetitive patterns of binding metal motifs in these structures. Several standard templates were parameterized and stored in a template library, which *metallicious* uses to parameterize input structures. Once *metallicious* identifies the template that matches the metal site in the structure, it copies the bonded parameters from the template and performs charge redistribution to account for charge transfer. To broaden the scope of the template library and increase efficiency, *metallicious* provides convenient truncation schemes, allowing for the recycling of available templates. In cases where no suitable template is found, *metallicious* automatically performs parameterization. The results of the benchmarks show good agreement with reference data obtained from QM and the crystal structure. While it can be argued that two other popular models, the nonbonded and cationic dummy atom model, offer more flexibility, the MD simulations conclusively show that the covalent metal model is the only robust option enabling simulations of these systems. Overall, *metallicious* provides a valuable resource for researchers working with metal-containing systems, facilitating their atomistic modeling in explicit solvent.

## Data Availability

The source code and associated Python files are freely available at https://github.com/duartegroup/metallicious. Documentation and tutorials are available at https://metallicious.readthedocs.io.

## Supporting Information Available

Implementation details, benchmark methods, and individual results (PDF)
MD topologies and input files (ZIP)
100 ns MD trajectories of the 11 supramolecular systems parameterized with *metallicious* and 3 supramolecular systems with the cationic dummy model (ZIP)
