# Supplementary material for: Metallicious: Automated Force-Field Parameterization of Covalently Bound Metals for Supramolecular Structures
Source: J Chem Theory Comput. 2024 Oct 7;20(20):9060–71. doi: 10.1021/acs.jctc.4c00850 (PMC11500408; doi:10.1021/acs.jctc.4c00850)
Supplement: Supplementary file 1 — ct4c00850_si_001.pdf [file ct4c00850_si_001.pdf]

Supporting information for:

metallicious: Automated force-field parametrization of covalently bound metals for supramolecular structures

Tomasz K. Piskorz,[1] Bernadette Lee,[1] Shaoqi Zhan,[1,2] Fernanda Duarte[1]*

[1]Department of Chemistry, University of Oxford, Oxford OX1 3QZ, UK.

[2]Department of Chemistry - Ångström, Ångströmlaboratoriet Box 523, S-751 20 Uppsala, Sweden

E-mail: fernanda.duartegonzalez@chem.ox.ac.uk

TABLE OF CONTENTS

S1. Execution of metallicious 3
S2. Details of implementation........ 4
S2.1. Parametrization using the template library 4
S2.1.1. Mapping of the template onto the metal site – a two-step process 4
S2.2. Parametrization of new template 5
S2.2.1. Partial charges........ 5
S2.2.2. Residual partial charges and truncation schemes........ 5
S2.2.3. Symmetrization of parameters for identical coordinating ligands........ 8
S3. Benchmark........ 10
S3.1. Systems........ 10
S3.2. Methods 11
S4. Additional results........ 13
S4.1. Inclusion of improper dihedrals........ 13
S4.2. Analysis of MD trajectories........ 14
References........ 29

S1. EXECUTION OF *METALLICIOUS*

Table S1. Input variables for *metallicious*.

Variable	Comment	Possible input	Default	Mandatory parameter?
-h, --help	show help message and exit	None	False	
-f	Metaloorganic coordination file	.gro, .pdb and other formats supported by MDAnalysis	None	Yes
-p	Metaloorganic topology	.top, .prmtop, etc. and other supported by ParmEd	None	Yes (unless prepare_initial_topology specified)
-of	Output metaloorganic structure	.gro, .pdb and other formats supported by MDAnalysis	out.pdb	No
-op	Output metaloorganic topology	.top, .prmtop and other formats supported by ParmEd	out.top	No
-metal_and_charges	Metal names and charges (optionally, multiplicity when parametrization needed)	Names and charges are separate by whitespace (e.g., Pd 2 Ru 2) or names, charges and multiplicities separated by spaces (e.g., Pd 2 1 Ru 2 1)	None	Yes
-keywords	autodE keywords for QM calculations	See autodE or ORCA manual/	PBE0 D3BJ def2-SVP tightOPT freq	No
-LJ_type	Type of parameters for Lennard-Jones paramters	uff, merz-tip3p, merz-opc3, merz-spc/e, merz-tip3p-fb, merz-opc, merz-tip4p-fb, merz-tip4-ew, zhang-tip3p, zhang-opc3, zhang-spc/e, zhang-spc/eb, zhang-tip3p-fb, zhang-opc, zhang-tip4p/2005, zhang-tip4p-d, zhang-tip4p-fb, zhang-tip4p-ew	Merz-opc	No
-truncate	Truncation scheme	None, 3bond/dihedral,	None,	No

		2bond/angle, 1bond/bond		
-improper_metal	Calculate the improper dihedral of the metal-aromatic	True/False	False	No
-donors	Donors from the connected ligands, usually electronegative atoms, such as N, S, O, but sometimes metal is connected to carbon	Any element name	N S O	No
-prepare_initial_topology	Prepare initial topology using GAFF	True/False	False	No
-linker_topol	Linker force-field (topology) parameters only used when prepare_initial_topology=True	.top, .prmtop, etc. and other supported by ParmEd	None	No
-rmsd_cutoff	Cutoff for the RMSD acceptance of the template	Float	2	No

S2. DETAILS OF IMPLEMENTATION

S2.1. Parametrization using the template library

S2.1.1. Mapping of the template onto the metal site – a two-step process

Mapping atoms from one structure to another can have a significant computational cost due to the need to identify all possible mappings (*i.e.*, isomorphic molecular graphs). For example, in the PdL_2L' complex (L=4-methylpyridine and L'=diamine, **Figure S1a**), the 4-methylpyridine ligand has only two possible mappings, while the diamine ligand has over 16,000 possible mappings (as calculated by NetworkX[1]). To reduce computational time, a two-step mapping process is used; first, the heavy atoms are mapped, followed by the hydrogen atoms, with heavy atoms constrained. This approach significantly reduces the number of permutations that need to be checked (**Figure S1b** and **S1c**).

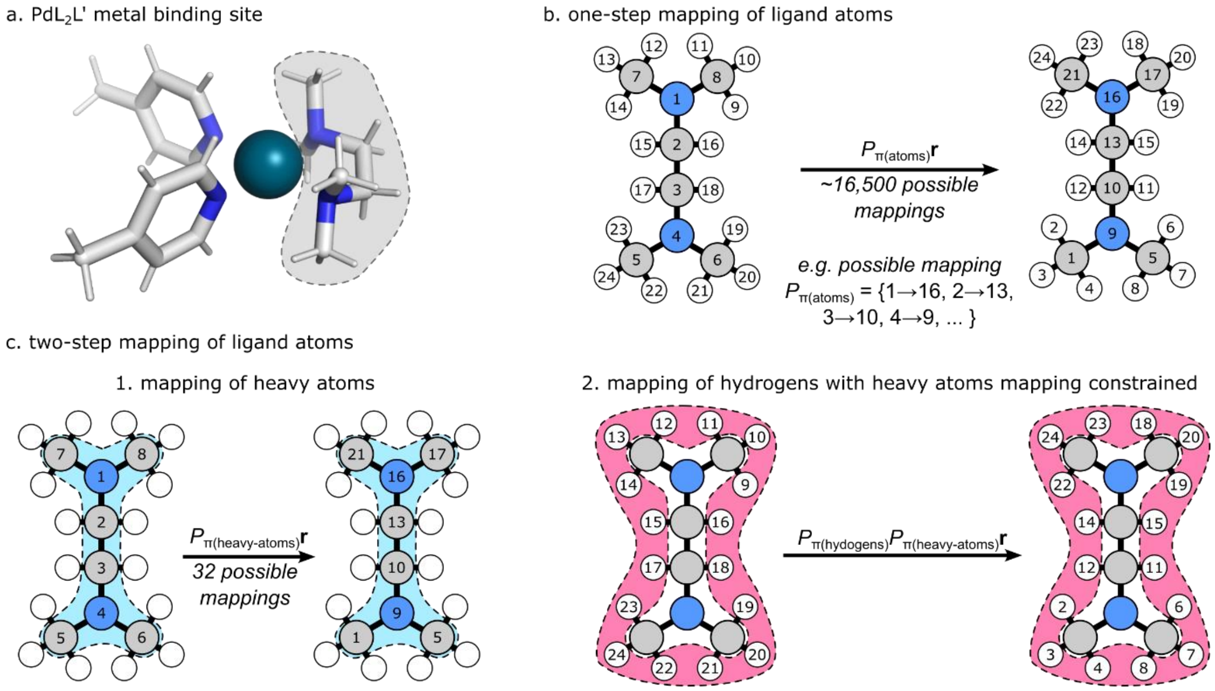

Figure S1. Mapping of ligand atoms between the template and metal binding site. (a) A PdL_2L' complex with L=4-methylpyridine and L'= diamine (highlighted in grey). (b) Mapping all atoms in the ligand results in a large number of possible mappings. (c) Mapping heavy atoms (highlighted by blue shading) first and then hydrogen atoms (highlighted by pink shading;) with heavy atoms constrained significantly reduces the number of permutations that need to be checked. P_π denotes permutation matrix.

S2.2. Parametrization of new template

S2.2.1. Partial charges

A RESP procedure was applied to obtain partial charges.[2] The conventional RESP procedure employs HF/6-31G*, which is known to overestimate the polarity of molecules in a vacuum, coincidently resulting in a polarisation similar to one anticipated in an aqueous solution.[2] On the other hand, B3LYP/6-31G*, popularly used for metal complexes, is limited to the first row of transition metals. To balance computational cost and robustness, *metallicious* uses D3BJ-PBE0/def2-SVP, which is available for elements up to the third row of the periodic table. Reassuringly, the RESP partial charges obtained with B3LYP/6-31G* and D3BJ-PBE0/def2-SVP level of theory show an insignificant difference (RMSE=0.01±0.0; **Figure S2)**.

S2.2.2. Residual partial charges and truncation schemes

To evaluate the quality of the computed charges in *metallicious*, we compared them to the charges those directly obtained from RESP at the D3BJ-PBE0/def2-SVP level of theory for small metal sites (**Figure S5**). In *metallicious*, partial charges are obtained as a sum of the input force field partial charges of ligands and metal ions and the residual partial charge of the template (**Figure S2a(ii)**). In the example shown in **Figure S2a(ii),** Fe has an initial force field charge of +2; however, it changes to 0.0 after the summation procedure. The RMSEs for all atoms of the obtained partial charges with the reference partial charges are shown in **Figure S2b.**

The partial charges obtained from *metallicious* were affected by the input force-field parameters. Specifically, the partial charges obtained from *metallicious* matched RESP charges better when the input ligands were parametrized with RESP charges (RMSE=0.08±0.04; **Figure S2b**) compared to when they were parametrized with AM1-BCC (RMSE=0.15±0.06; **Figure S2b**). The quality of partial

charges was also evaluated for different truncation schemes. As expected, the quality of reproduced charges deteriorates with increased truncation (RMSE=0.14±0.06 for 3-bond truncation, RMSE=0.16±0.04 for 2-bond truncation, RMSE=0.19±0.02 for 1-bond truncation; **Figure S2b**).

As anticipated, the partial charges obtained from non-bonded model deviated from RESP significantly (RMSE=0.35±0.05; **Figure S2b**). This is expected as charge-transfer was not considered in the non-bonded model.

We also investigated the impact of incorporating electron-withdrawing (EWG, $-NO_2$) and electron-donating groups (EDG, $-NH_2$) into the aromatic groups bound to the metal on partial charges (**Figure S3**). We observed only a slight deterioration in reproducing the reference partial charges after summation compared to the results from the non-functionalized systems, RMSE=0.17±0.05 for EWG/EDG set *vs.* 0.15±0.06 for non-functionalized systems (**Figure S2b**). Thus, confirming the transferability of this approach.

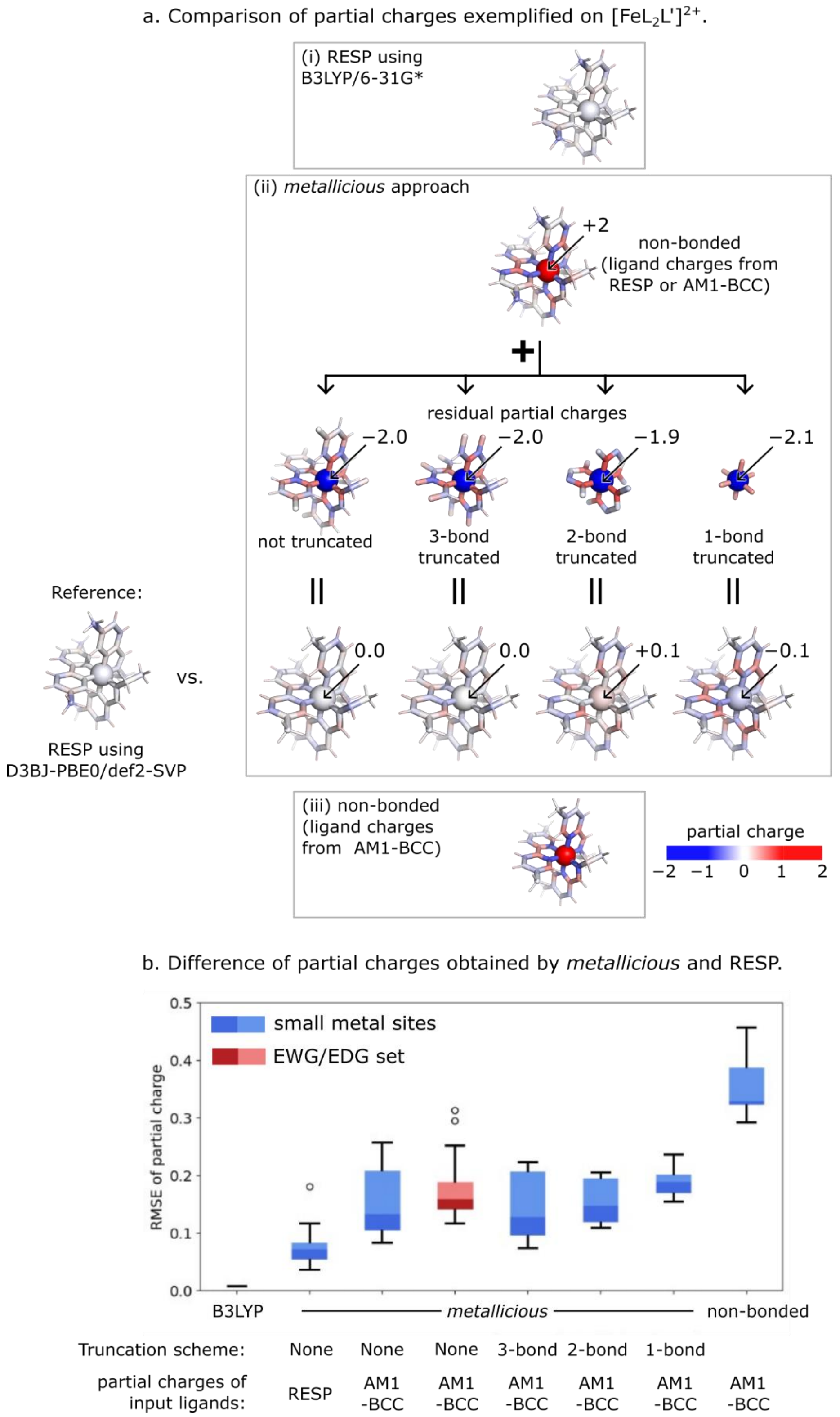

Figure S2. a) Partial charges for $[FeL_2L']^{2+}$(L=(E)-N-ethyl-1-(5-methylpyridin-2-yl)methanimine, L'=abametapir) complex obtained with different methods. Reference partial charges were obtained using RESP at the D3BJ-PBE0/def2-SVP level of theory. They are compared to (i) RESP at B3LYP/6-31G* level of theory, (ii) *metallicious* obtained charges from non-bonded model + residual partial charges (with none, 3-bond, 2-bond and 1-bond truncation scheme) and (iii) non-bonded model with partial charges of ligands' atoms calculated using AM1-BCC and metal's charge as integer charge, +2. b) RMSE of partial charges obtained from RESP method at B3LYP/6-31G* level of theory, from *metallicious* (using different templates) and non-bonded model for the 11 small metal sites (**Table S2, Figure S5**) relative to RESP at D3BJ-PBE0/def2-SVP level of theory. Blue-shaded colours represent results for small metal sites, while red-shaded colours represent the results for small metal sites functionalized with electron-withdrawing ($-NO_2$) and electron-donating ($-NH_3$) groups (EWG/EDG).

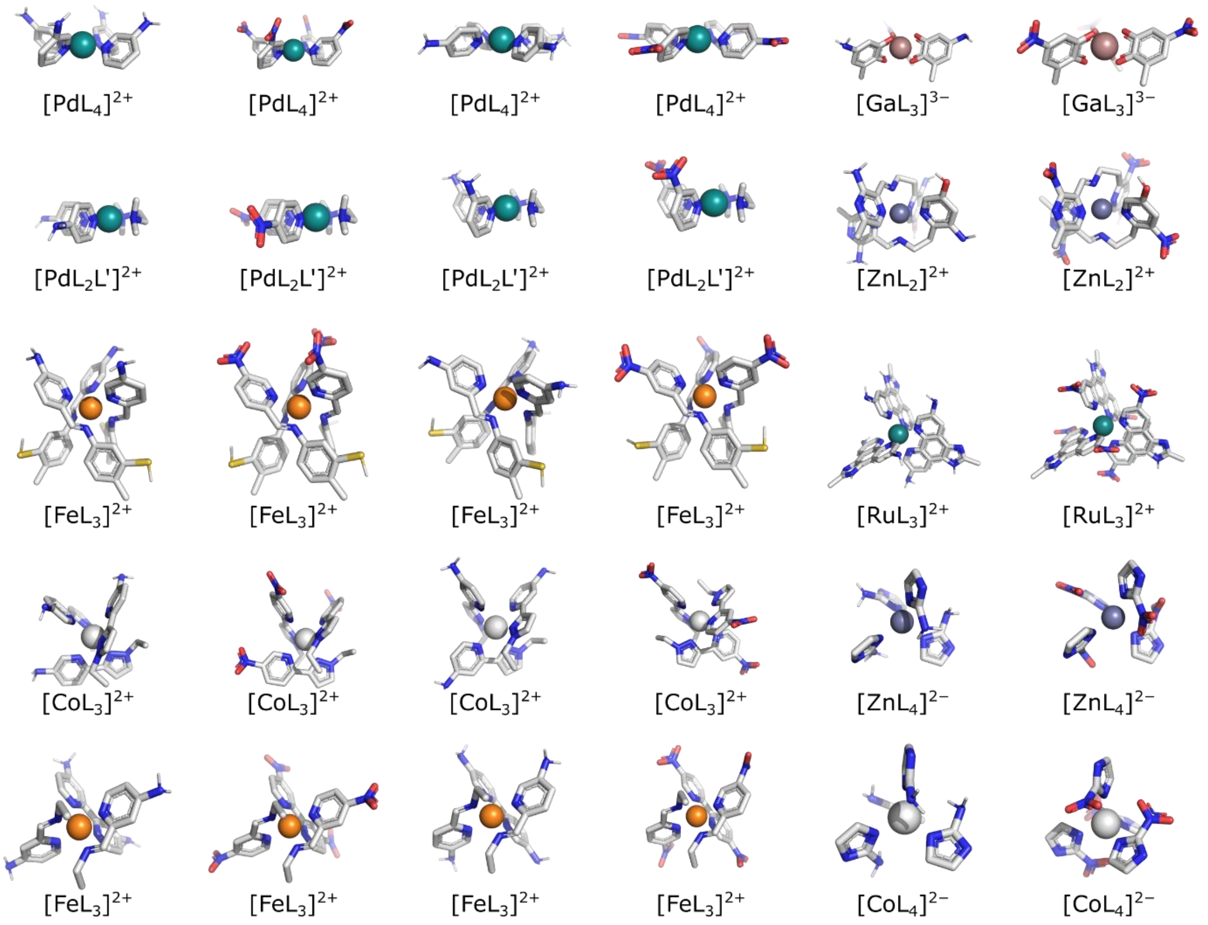

Figure S3. Structures of small metal sites functionalized with electron-withdrawing (nitro groups) and electron-donating (amino groups) groups.

S2.2.3. Symmetrization of parameters for identical coordinating ligands

When the ligands in a template are identical, their bonded parameters should also be identical. This can be achieved by symmetrizing the parameters through mapping between two molecular graphs. Including the metal in this process allows metal-ligand bonds to be part of the symmetrization (**Figure S4a**), resulting in identical bonded parameters and partial charges for identical ligands. This mapping symmetrizes most bonded interactions, except for angles connecting two different ligands via the metal. In many cases, such as a square planar $[PdL_4]^{2+}$ complex (L=Me-pyridine), ligand arrangements can seem identical in molecular graph representation, but their angles N-Pd-N have different values in the structure ($\angle$(N-Pd-N)$_{trans}$=180° and $\angle$(N-Pd-N)$_{cis}$=90°) such that those parameters are not identical and therefore cannot be symmetrized (**Figure S4b**).

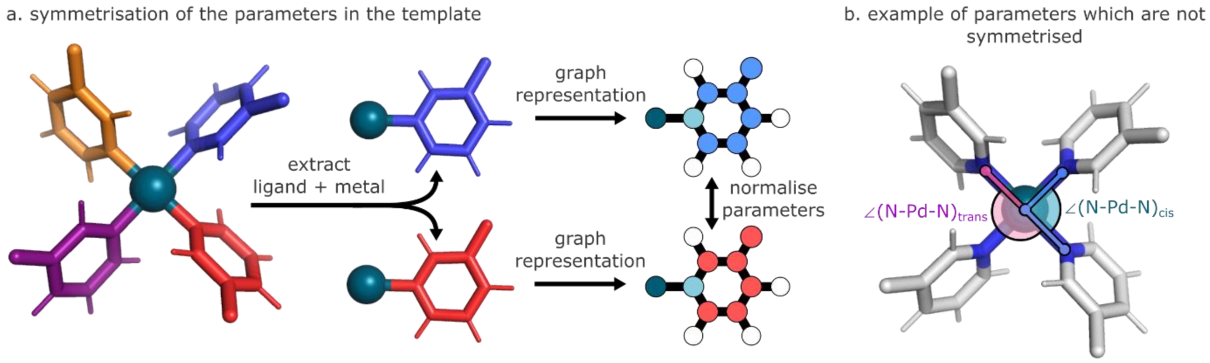

Figure S4. (a) Symmetrization procedure for identical ligands in a template, *e.g.* $[PdL_4]^{2+}$ complex (L=Me-pyridine). (b) Example of angle parameters, ∠N-Pd-N, involving metal that are not symmetrized as they are identical in a molecular graph representation but have different values in a 3D structure ($\angle(N\text{-}Pd\text{-}N)_{trans}$=180° (pink), and $\angle(N\text{-}Pd\text{-}N)_{cis}$=90° (blue)).

S3. BENCHMARK

S3.1. Systems

The benchmark includes 11 systems whose structures were obtained from the Cambridge Crystallographic Data Centre (CCDC) (**Table S2, Figure S4)**. From these structures, saturated templates were extracted (**Figure 6a** in the main text), forming small metal sites benchmark set.

Table S2. Small metal sites extracted from the corresponding structures available in the CCDC, listed below. Their structures are shown in **Figure S5**. DCM= dichloromethane; DMSO=dimethylsulfoxide; MeOH=methanol; MeCN=acetonitrile, OTf^{-}= triflate, NMe_4^{+}= tetramethylammonium, NO_3^{-}= nitrate, BF_4^{-}= tetrafluoroborate.

Id	Structure	CCDC	Solvent	Anion	Reference
1	$[Pd_2L_4]^{4+}$	1492902	DCM	OTf^{-}	3
2	$[Ga_4L_6]^{12-}$	718471	H_2O	K^{+}	4
3	$[Fe_4L_6]^{4-}$	727817	H_2O	NMe_4^{+}	5
4	$[Pd_6L_4]^{12+}$	1045836	H_2O	NO_3^{-}	6
5	$[Co_8L_{12}]^{16+}$	1569921	MeOH	BF_4^{-}	7
6	$[Pd_6Ru_8L_{24}]^{28+}$	1432349	DMSO	BF_4^{-}	8
7	$[Pd_{48}L_{96}]^{96+}$	1831431	DMSO	BF_4^{-}	9
8	$[Fe_5L_5]^{10+}$	875091	DMSO	Cl^{-}	10
9	$[Zn_3L_3]^{6+}$	1815058	MeCN	BF_4^{-}	11
10	ZIF-8	864311	None	None	12
11	ZIF-67	671073	None	None	13

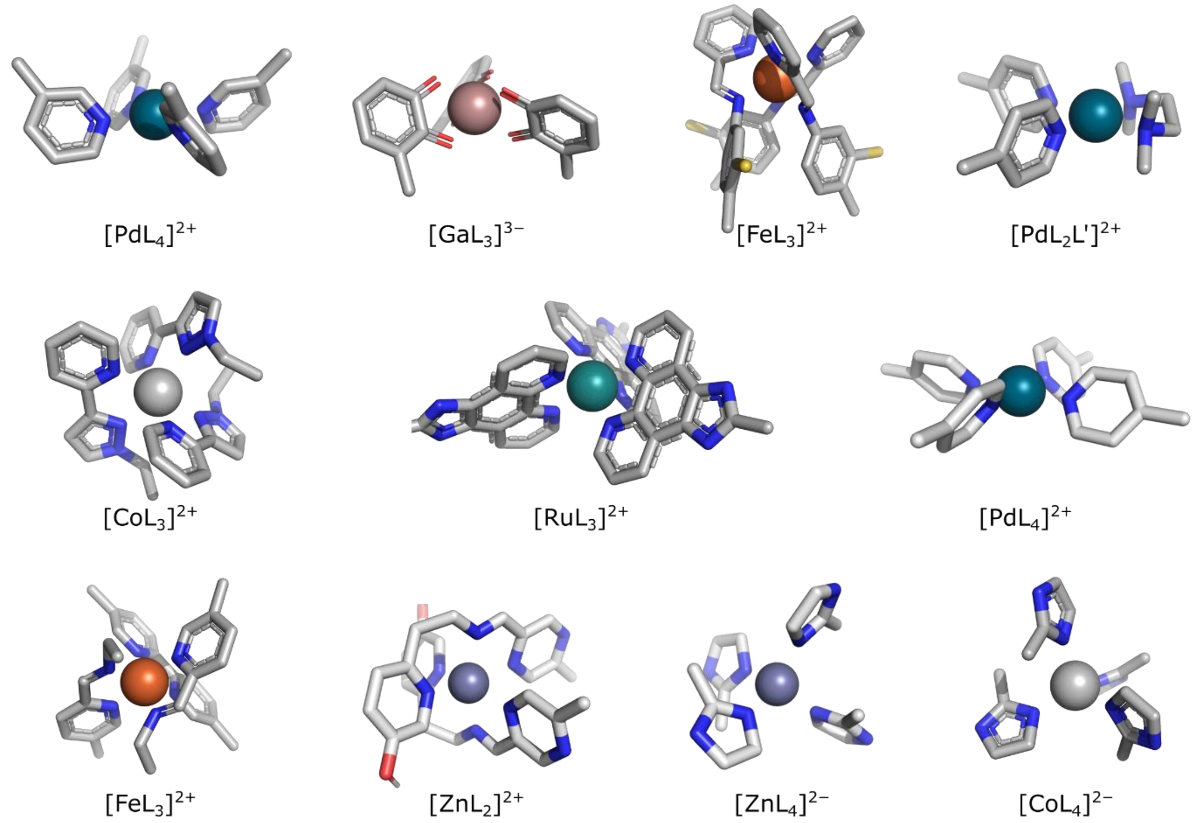

Figure S5. Structure of small metal sites used in this study for benchmarking, obtained by template extraction from crystal structures obtained from CCSD.

S3.2. Methods

S3.2.1. Structures and frequency calculations of saturated templates The molecular mechanics (MM) normal modes and frequencies were computed using GROMACS 2021.3 with double-precision, required for normal model analysis.[14] The Broyden–Fletcher–Goldfarb–Shanno (BFGS) algorithm was used for optimization until the maximum force was smaller than 10^{-8} kJ mol^{-1} nm^{-1}. Comparison to quantum mechanics (QM) computed values, considered "ideal" was performed similarly to the method used by Allen *et al.* computing the mean percentage error as follow:

$$\text{Error: } = \frac{100}{3N-6}\sum_{i=1}^{3N}\left|\frac{\alpha\nu_i^{QM}-\nu_i^{\nu\,MM}}{\alpha\nu_i^{QM}}\right| \quad \text{(Eq. S1)},$$

where ν_i^{QM} and ν_i^{MM} are the frequencies obtained from *i*-th QM and MM normal mode, respectively, α is the vibrational scaling factor, and *N* is the number of atoms in the molecule.[15] Moreover, the MM optimized structures were compared to those obtained from QM calculations using RMSD.

S3.2.2. Comparison of MM computed and crystal structure

The structures obtained from CCDC were parametrized using *metallicious*. The ZIF-8 and ZIF-67 structures were optimized with double-precision GROMACS with periodic boundary conditions in a vacuum. The rest of the structures were optimized using AMBER software without periodic boundary conditions using OBC implicit model,[16] with dielectric constant for the corresponding solvent. They

included dichloromethane (DCM: 8.93), dimethylsulfoxide (DMSO: 47.2), methanol (MeOH: 32.7), acetonitrile (MeCN: 36.6), and water (H_2O: 78.4).

S3.2.3. Binding energy of benzoquinone bound to $[Pd_2L_4]^{4+}$

The MM binding energy of benzoquinone (**bq**) bound to $[Pd_2L_4]^{4+}$ was calculated using AMBER software and the OBC implicit model of DCM (ε = 8.93). **bq** was placed at the centre of the cage with its oxygens aligned with the metal centers. Then, the system was energy minimized for 5,0000 steps using limited-memory BFGS method. Similar calculations were performed for the separate cage and **bq**. The binding energy was determined by calculating the difference between the final MM energies of the (**bq**) $\subset[Pd_2L_4]^{4+}$ complex and the separate species, **bq** and $[Pd_2L_4]^{4+}$. The reference binding energy was obtained at the CPCM(DCM)-M06-2X/def2-TZVP level of theory (**Table S3**).

Table S3. Binding energy of **bq** in $[Pd_2L_4]^{4+}$ calculated at the CPCM(DCM)-M06-2X/def2-TZVP//CPCM(DCM)-PBE0-D3BJ/def2-SVP level of theory.

Species	Absolute energy [Eh]
**bq**	-381.4550
$[Pd_2L_4]^{4+}$	-3770.0087
**bq** $\subset[Pd_2L_4]^{4+}$	-4151.4805
ΔE [kcal mol^{-1}]	-10.6

Molecular Dynamics (MD) simulations. MD simulations were performed using GROMACS 2021.3 (single precision).[14,17] Water was modelled using OPC parameters,[18] while parameters for organic solvents, DCM, DMSO, MeOH, and MeCN were taken from VirtualChemistry.org,[19] Organic molecules and anions were parametrized using Antechamber with GAFF parameters.[20] The temperature was kept constant at 300 K using the v-rescale[21] thermostat, while pressure was fixed at 1 bar using the c-rescale barostat.[22] Long-range electrostatic interactions were accounted for using the smooth particle mesh Ewald (PME) method.[23,24] A cut-off of 1.0 nm was used for van der Waals interactions and short-range electrostatics.

Each supramolecular system, with the exception of ZIF-8 and ZIF-6, was placed in a cubic box with a 1.5 nm distance between the solute and the edge of the box. The systems were then neutralized and solvated using the ions and solvent experimentally reported (**Table S2**).

For the simulation of periodic MOFs, the crystal structures available for ZIF-8 and ZIF-67 (CCDC 864311 and CCDC 671073, respectively) was used as the starting configuration. The unit cell was replicated four times along each axis to ensure the system size exceeded the cut-off for electrostatic interactions.

Simulations were initiated with energy minimization followed by 20 ps of NVT equilibration and 20 ps of NPT simulation, using 2fs step size. Production runs were performed for 100 ns. For simulations involving a dummy metal model, a 1 fs timestep was used to ensure the stability of the system. Each system was run with a single replica.

S4. ADDITIONAL RESULTS

S4.1. Inclusion of improper dihedrals.

To assess the impact of including metal-involving improper parameters on the quality of the structure, we optimized all structures with and without these parameters in implicit solvent. While adding these parameters improved the stability of some structures, as shown by RMSD relative to the crystal structure (**Figure S6a**), others remained unaffected. Notably, these parameters were key for structures containing square planar palladium cations, $[Pd_2L_4]^{4+}$, $[Pd_6Ru_8L_{24}]^{28+}$, and $[Pd_{48}L_{96}]^{96+}$ (**Figure S6b**). Interestingly, $[Pd_6L_4]^{12+}$, is unaffected by metal-involving improper parametrization likely due to constraints imposed by the ligands (**Figure S5b**).

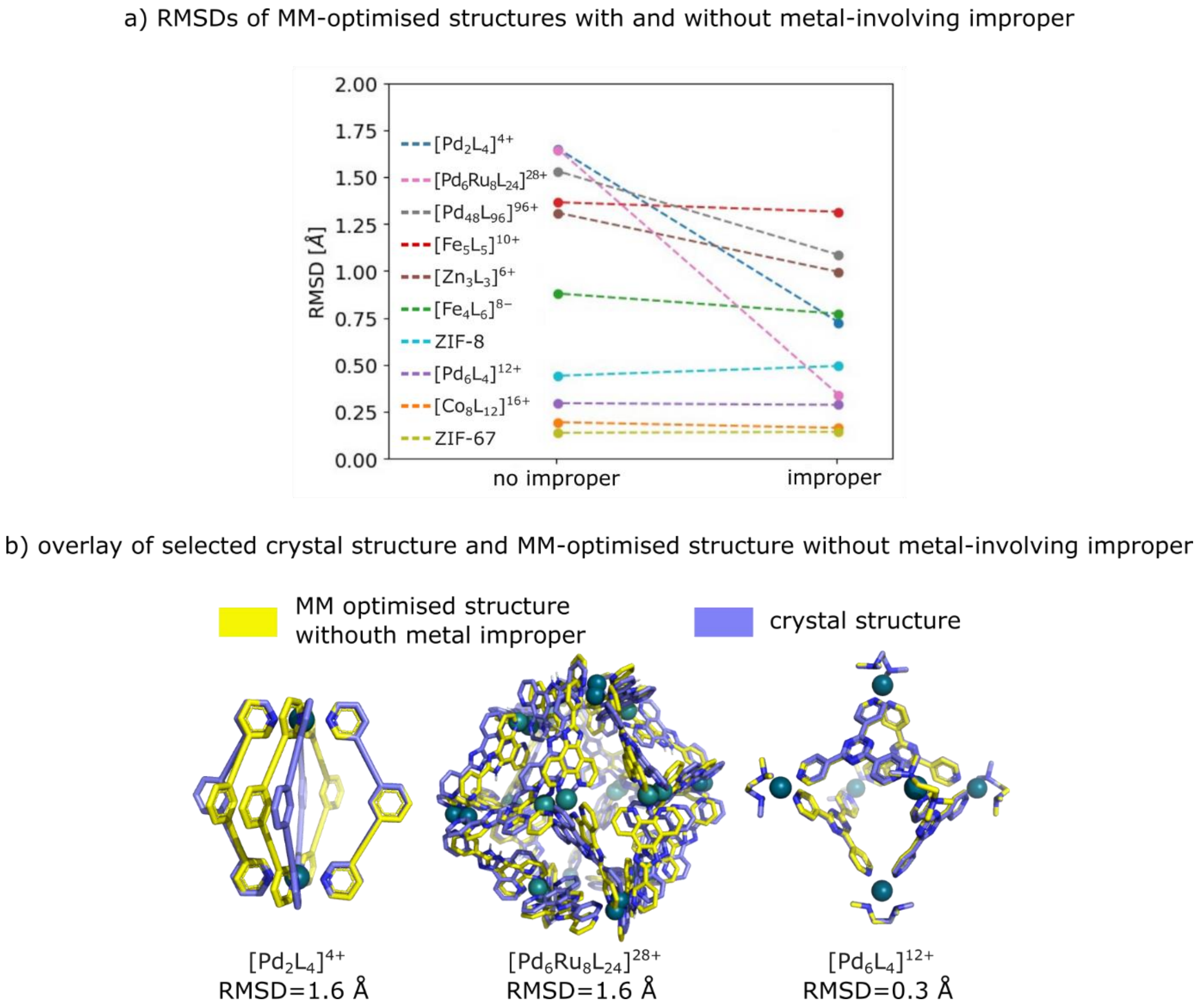

Figure S6. Importance of metal-involving improper dihedral angles. (a) RMSDs of structures optimized with and without the improper dihedral. (b) Overlay of selected snapshots of structures optimized without metal-involving improper dihedral with their respective crystal structure.

S4.2. Analysis of MD trajectories

S4.2.1. Stability of supramolecular systems.

We analyzed the stability of the supramolecular structures by evaluating the number of ligands bound to the metal during the simulation. This was calculated as the average number of donor atoms within 3 Å of the metal centres divided by ligand's denticity (**Table S4** and **Figures S6-S16**). A structure was considered stable if the number of coordinating ligands remained constant throughout the simulation (**Figure S6-S16b**). Additionally, the RMSD of heavy atoms of the supramolecular structures was calculated relative to the crystal structure using MDAnalysis (**Figures S6-S16c**).[25,26]

Previous works by Yoneya et al. have explored the self-assembly and dynamics of Pd-cages employing Langevin (stochastic) dynamics and using a cationic dummy model to represent the metal and a united-atom potential to describe the ligand.[27,28] Solvent effects were accounted for using the generalized reaction field method.[29] Our analysis indicates that the poor performance of the cation dummy model in our benchmark can be attributed to both solvent and counterions effects. When described explicitly, these effects compete with the metal-ligand interactions driving assembly. In the benchmark, we performed simulations $[Pd_2L_4]^{4+}$, $[Pd_6L_4]^{12+}$ and $[Pd_{48}L_{96}]^{96+}$ in the solvents reported in the literature, that is, DCM,[3] water,[6] and DMSO,[9] respectively. We observed that the only stable simulation was in DCM without counterions. The cationic dummy model performs well in such a scenario due to the lack of competitive interactions. In contrast, metal-solvent interactions compete with metal-ligand interactions when the system is modelled in polar solvents, such as water or DMSO. Indeed, $[Pd_2L_4]^{4+}$ simulated in explicit DMSO solvent without anions disassembles, while $[Pd_4L_6]^{12+}$ and $[Pd_{48}L_{96}]^{96+}$ simulated in explicit DCM maintain stability through simulation (**Figure S18**). This is in line with the necessary fine-tuning of the solvent dielectric reported by Yoneya et al.[27,28]

In the simulations conducted for MOF in the NPT ensemble we evaluated the change of unit-cell parameters during the simulation, as they are known to be sensitive to the application of pressure.[30] We did not observe significant changes, especially with the covalent model obtained from *metallicious* (**Table S5**).

Table S4. Summary of stability of the simulated systems. The systems were considered stable when no change in coordination sphere was observed (green). Conversely, a decrease in the coordination number was considered to correspond to an unstable system (blue). Simulations that were impossible due to the lack of the dummy model parameters are highlighted in grey.

**System**	**Non-bonded**	**Non-bonded w/o ions**	**Dummy model**	**Dummy model w/o ions**	***metallicious***
$[Pd_2L_4]^{4+}$	Unstable	Stable	Unstable	Stable	Stable
$[Ga_4L_6]^{12-}$	Unstable	Unstable	N/A	N/A	Stable
$[Fe_4L_6]^{4-}$	Stable	Unstable	Unstable	Unstable	Stable
$[Pd_6L_4]^{12+}$	Unstable	Unstable	Unstable	Unstable	Stable
$[Co_8L_{12}]^{16+}$	Unstable	Stable	Unstable		Stable
$[Pd_6Ru_8L_{24}]^{28+}$	Unstable	Unstable	N/A	N/A	Stable
$[Pd_{48}L_{96}]^{96+}$	Unstable	Unstable	Unstable	Unstable	Stable
$[Fe_5L_5]^{10+}$	Unstable	Unstable	Unstable	Unstable	Stable
$[Zn_3L_3]^{6+}$	Unstable	Stable	Unstable	Stable	Stable
ZIF-8	Stable	Stable	Stable	Stable	Stable
ZIF-67	Stable	Stable	Stable	Stable	Stable

Table S5. Change of the unit-cell parameters after 100 ns MD simulation for the ZIF-8 and ZIF-67 system in reference to crystal structure.

		a=b=c [Å]	α=β=γ [°]	Relative change [%]
ZIF-8	Crystal	58.86	109.47	
	Non-bonded	57.27	109.47	2.7
	Cationic dummy model	59.25	109.47	0.7
	metallicious	58.79	109.47	0.1
ZIF-67	Crystal	58.75	109.47	
	Non-bonded	58.06	109.47	1.2
	metallicious	58.52	109.47	0.4

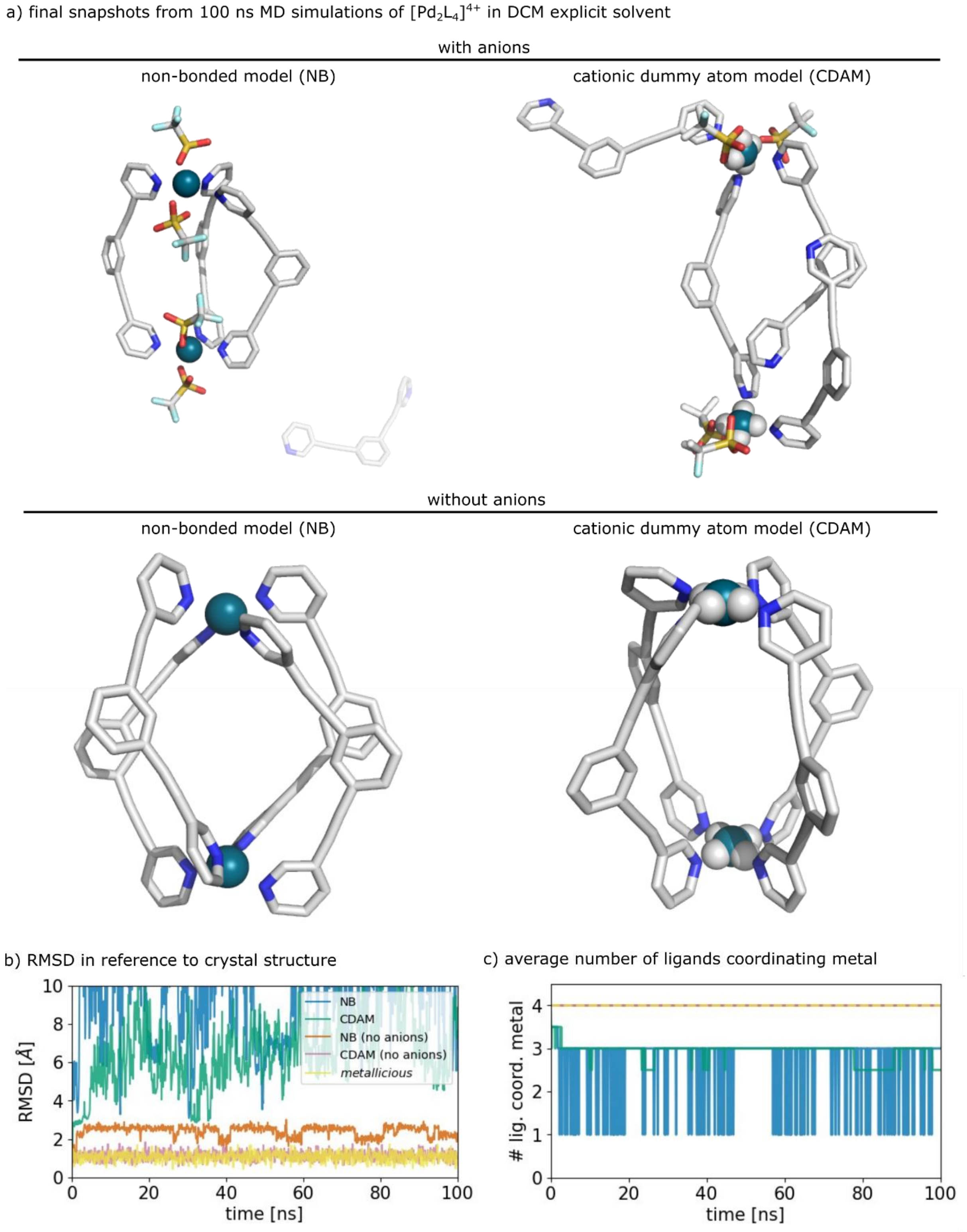

Figure S7. MD simulations of $[Pd_2L_4]^{4+}$ cage in explicit DCM. (a) Final snapshots for non-bonded (NB) model and cationic dummy atom model (CDAM) of metals with and without triflate **(**OTf^-) anions; solvent not shown for clarity. (b) RMSD relative to starting structure for NB model (blue), CDAM (green), NB model without anions (orange), CDAM without anions (pink) and *metallicious* (yellow). (c) Average number of ligands coordinating metals.

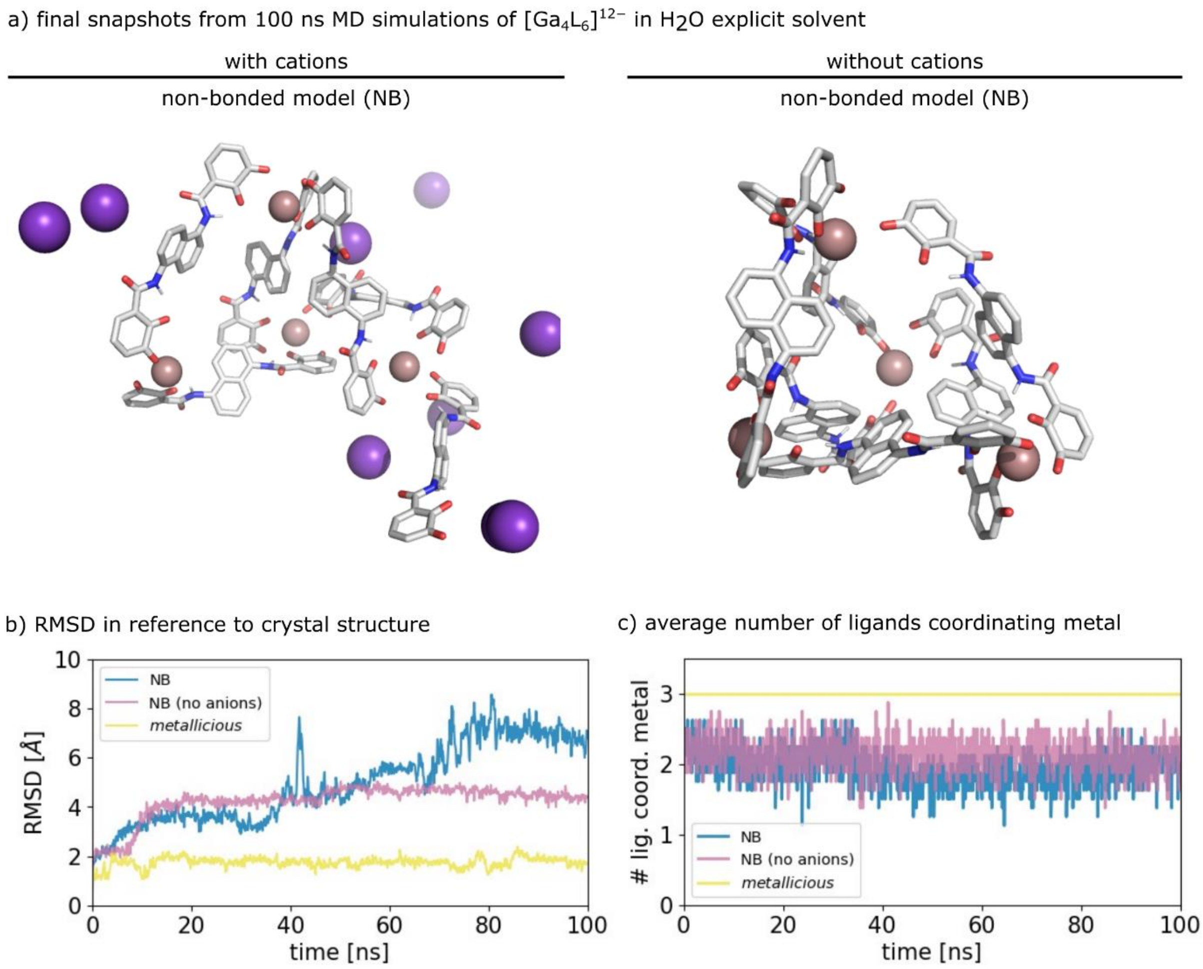

Figure S8. MD simulations of $[Ga_4L_6]^{12-}$ cage in explicit OPC water model. (a) Final snapshots of a non-bonded (NB) metal model with and without K^+ cations (purple balls); solvent not shown for clarity. (b) RMSD relative to starting structure for NB model with (blue) and without K^+ cations (purple), and *metallicious* (yellow). (c) Average ligand-metal coordinating number.

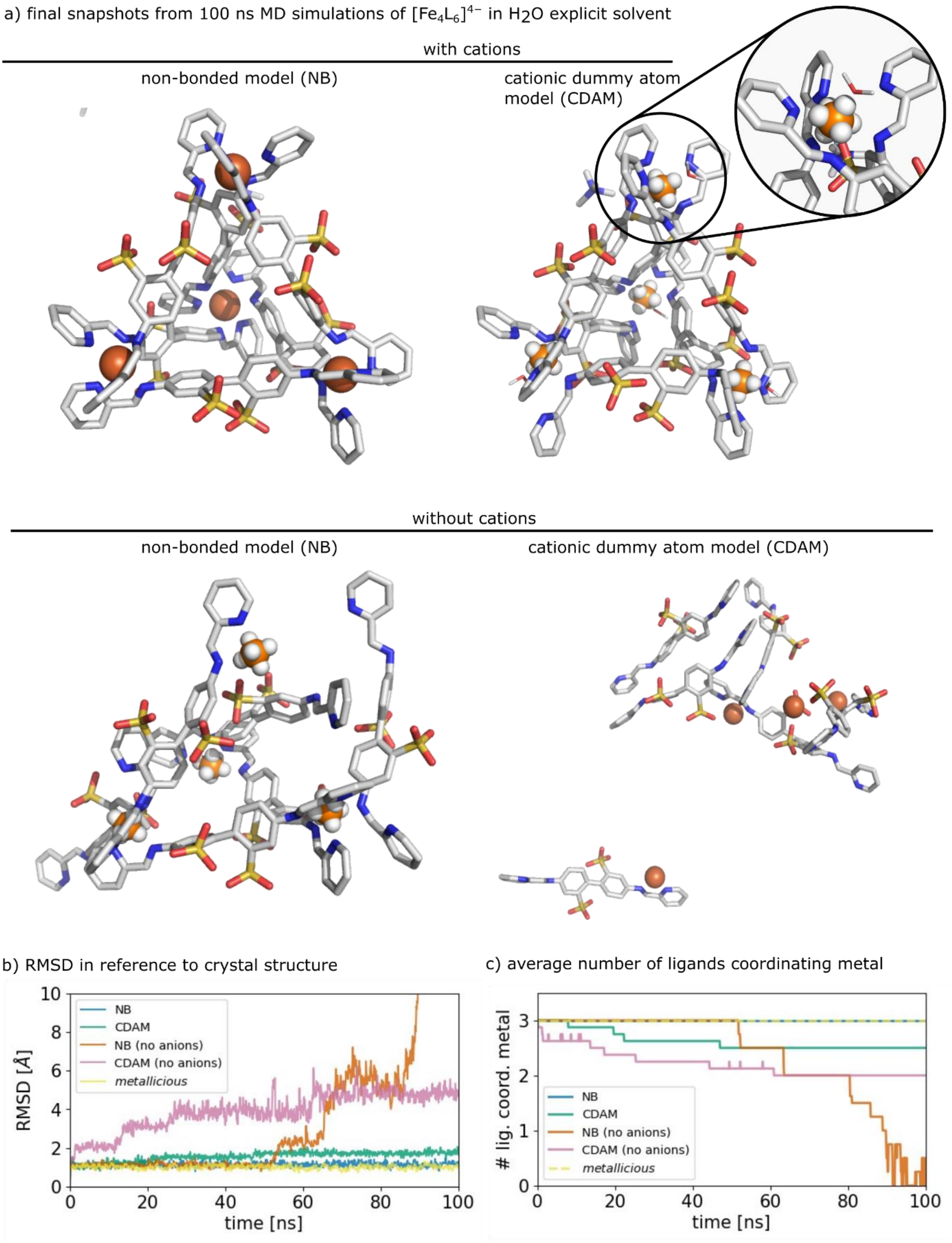

Figure S9. MD simulations of $[Fe_4L_6]^{4-}$ cage in explicit OPC water model. (a) Final snapshots for non-bonded (NB) and cationic dummy atom model (CDAM) of metals with and without NMe_4^+ cations (solvent not shown for clarity). (b) RMSD relative to starting structure for NB model (blue), CDAM (green), NB model without NMe_4^+ cation (orange), CDAM without NMe_4^+ cations (purple) and *metallicious* (yellow). (c) Average number of ligands coordinating metals.

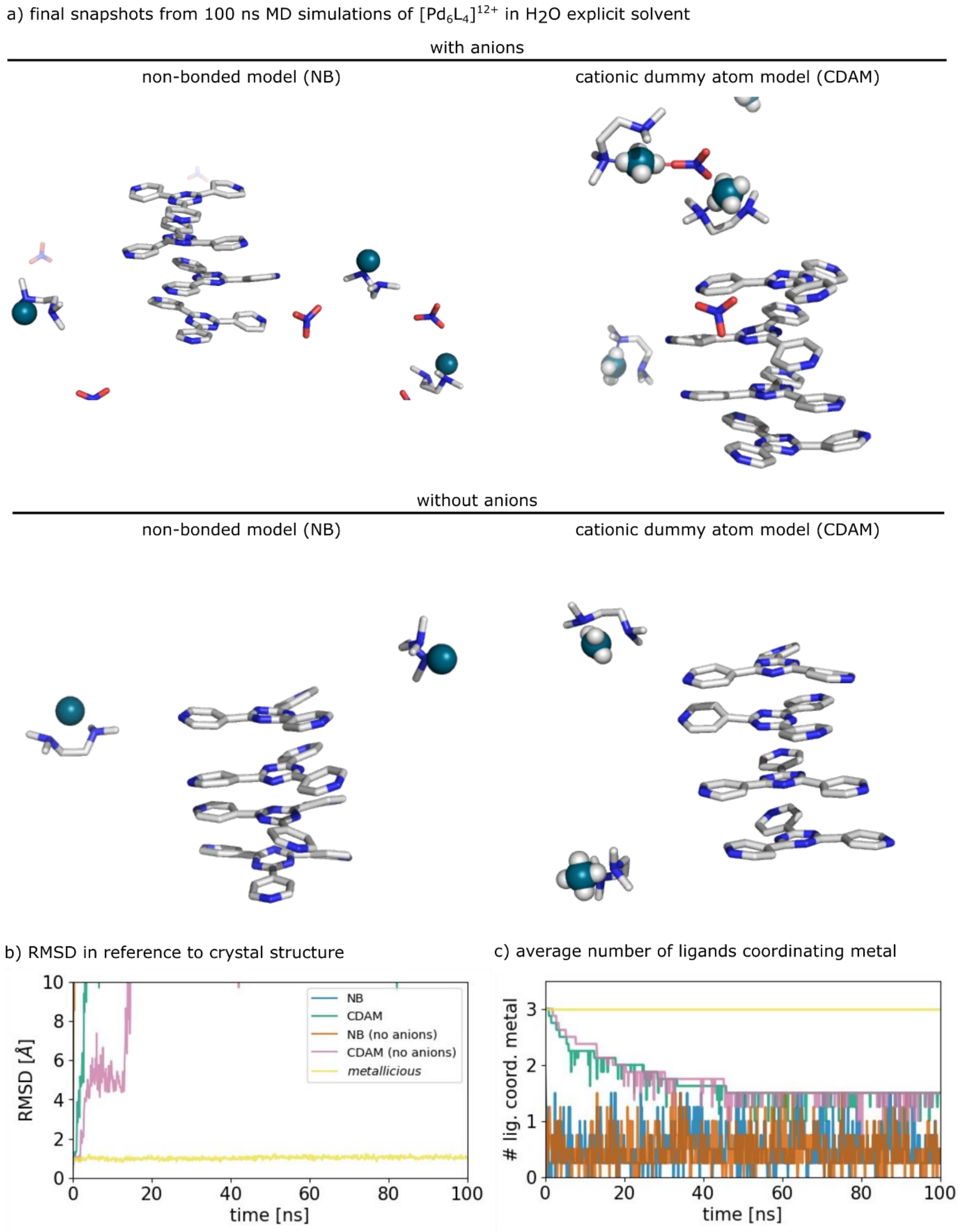

Figure S10. MD simulations of $[Pd_6L_4]^{12+}$ cage in explicit OPC water model. (a) Final snapshots for non-bonded (NB) model and cationic dummy atom model (CDAM) of metals with and without NO_3^- anions (solvent not shown for clarity). (b) RMSD relative to starting structure for NB model (blue), CDAM (green), NB model without anions (orange), CDAM without anions (pink) and *metallicious* (yellow). (c) Average number of ligands coordinating metals.

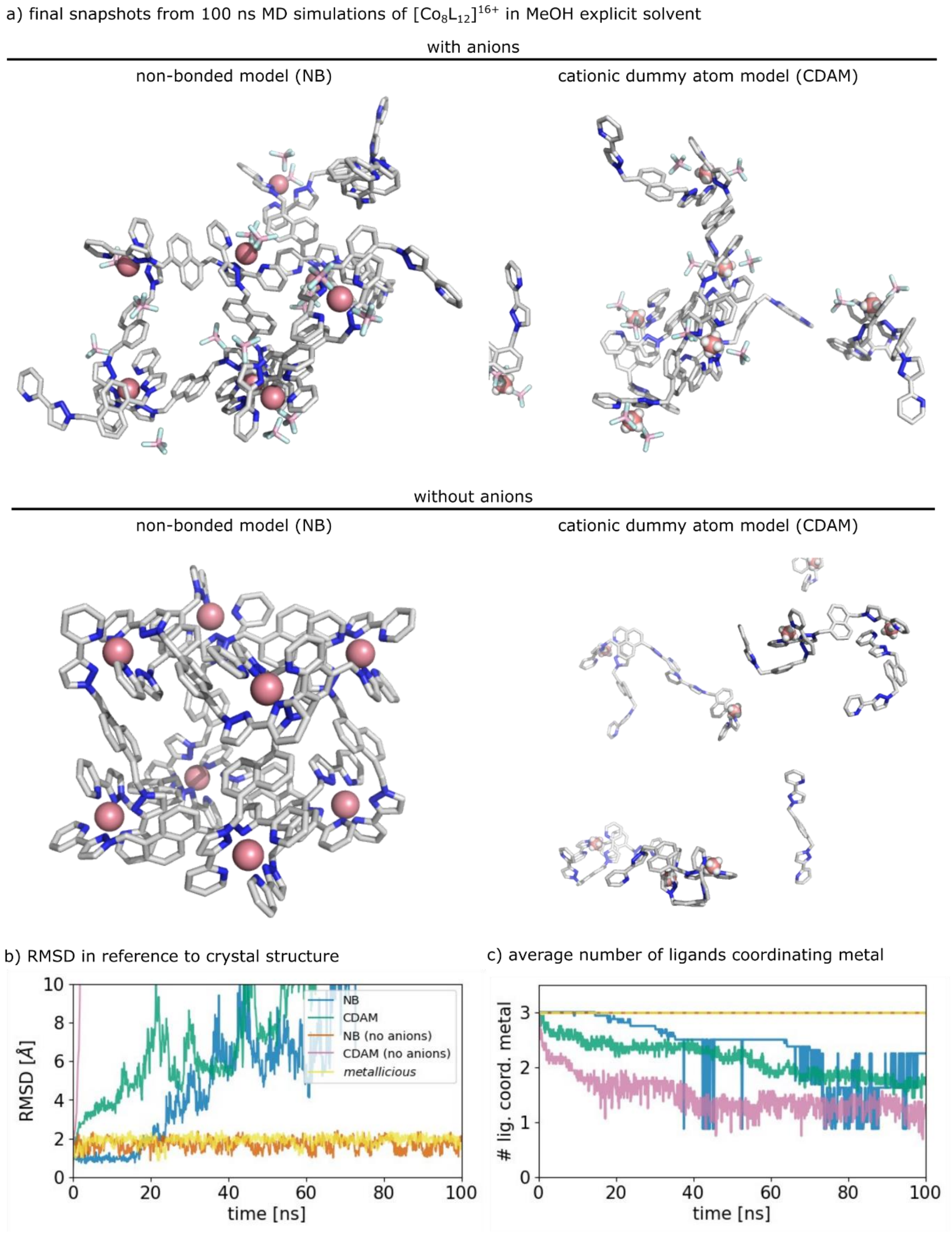

Figure S11. MD simulations of $[Co_8L_{12}]^{16+}$ cage in explicit MeOH. (a) Final snapshots for non-bonded (NB) model and cationic dummy atom model (CDAM) of metals with and without BF_4^- (solvent not shown for clarity). (b) RMSD relative to starting structure for NB model (blue), CDAM (green), NB model without anions (orange), CDAM without anions (purple) and *metallicious* (yellow). (c) Average number of ligands coordinating metals.

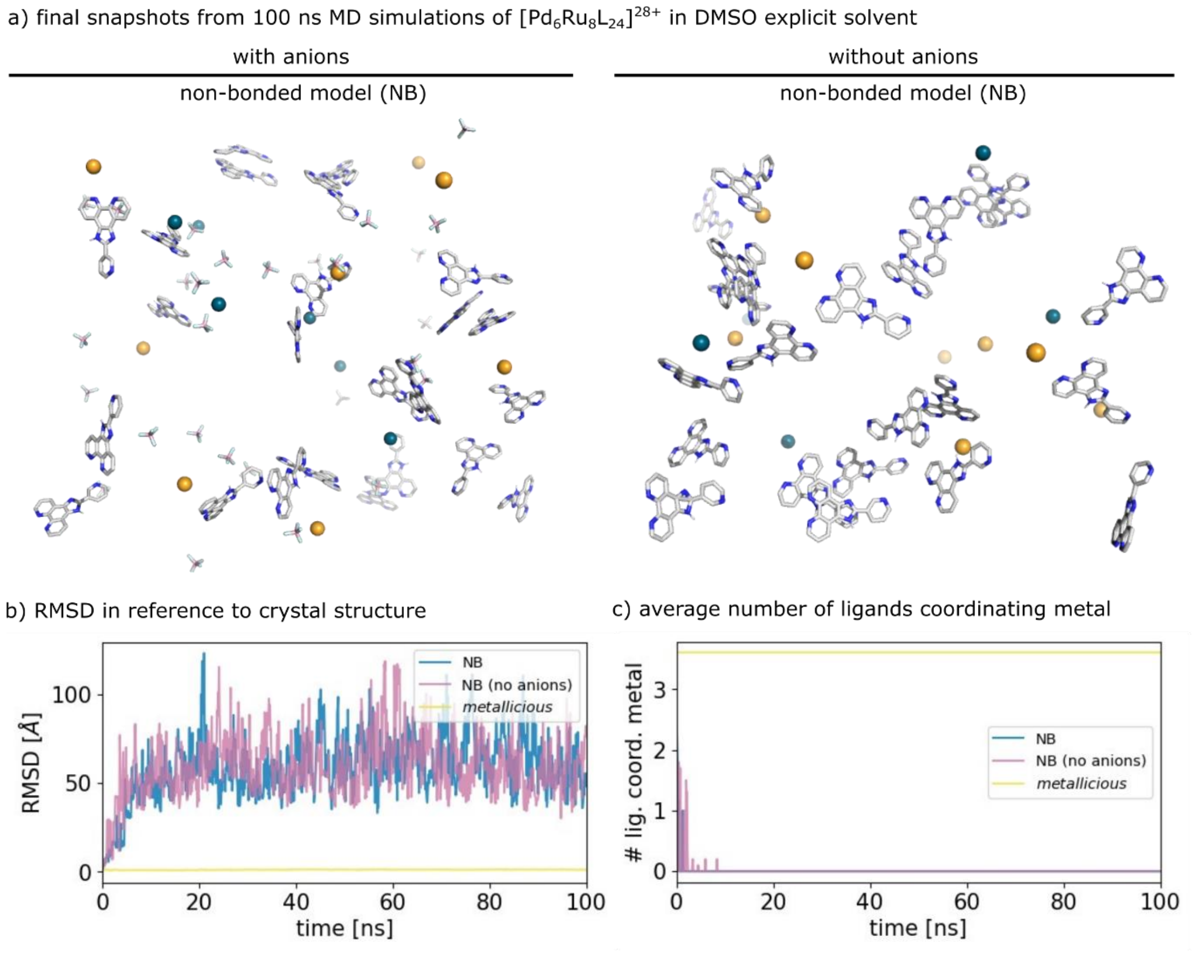

Figure S12. MD simulations of $[Pd_6Ru_8L_{24}]^{28+}$ cage in explicit DMSO. (a) Final snapshots for non-bonded (NB) model of metals with and without BF_4^- anions; Pd^{2+} as teal balls and Ru^{2+} as orange balls, solvent not shown for clarity. (b) RMSD relative to starting structure for NB model (blue), NB model without anions (purple) and *metallicious* (yellow). (c) Average number of ligands coordinating metals.

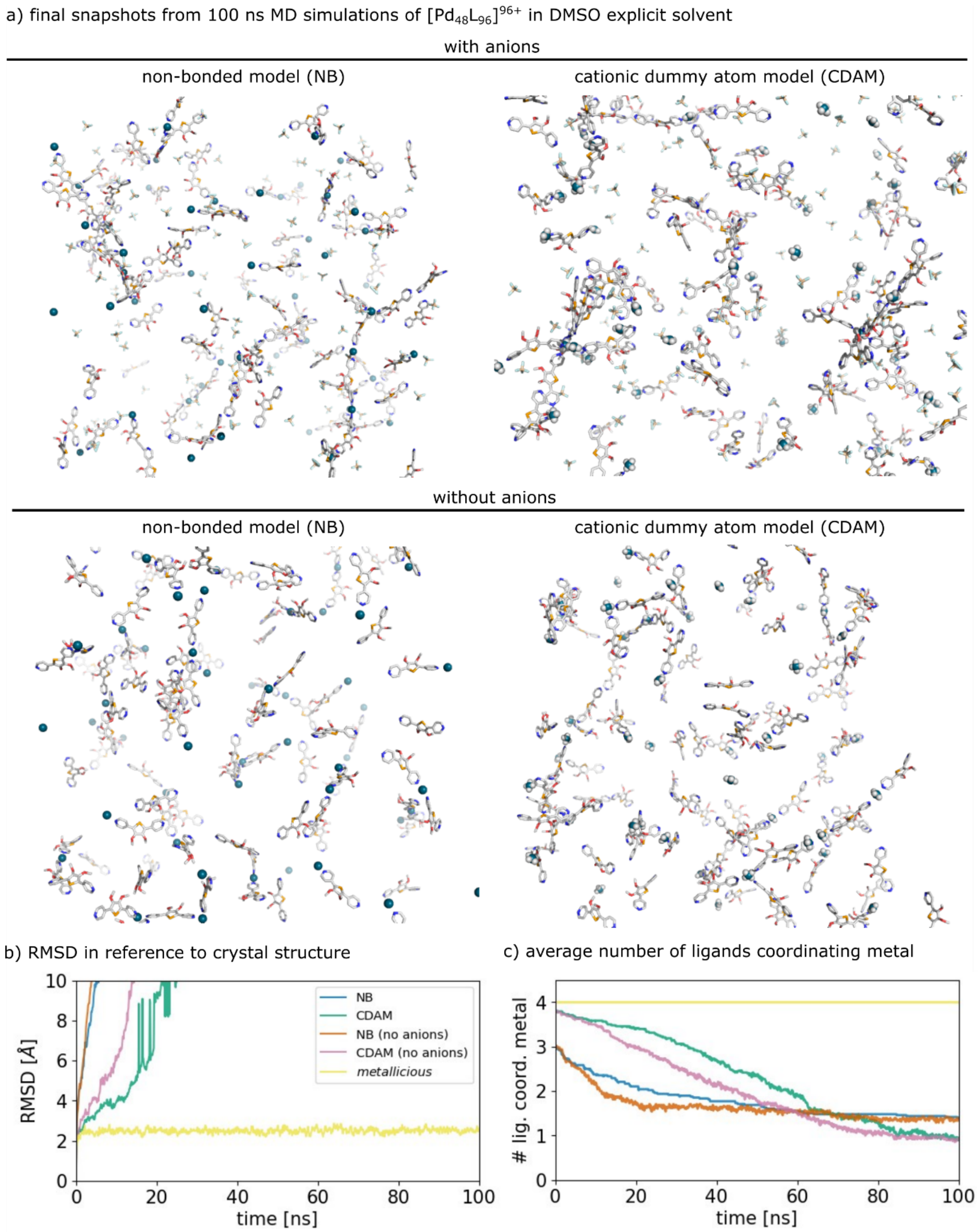

Figure S13. MD simulations of $[Pd_{48}L_{96}]^{96+}$ cage in explicit DMSO. (a) Final snapshots for non-bonded (NB) model and cationic dummy atom model (CDAM) of metals with and without BF_4^- anions (solvent not shown for clarity). (b) RMSD relative to starting structure for NB model (blue), CDAM (green), NB model without anions (orange), CDAM without anions (purple) and *metallicious* (yellow). (c) Average number of ligands coordinating metals.

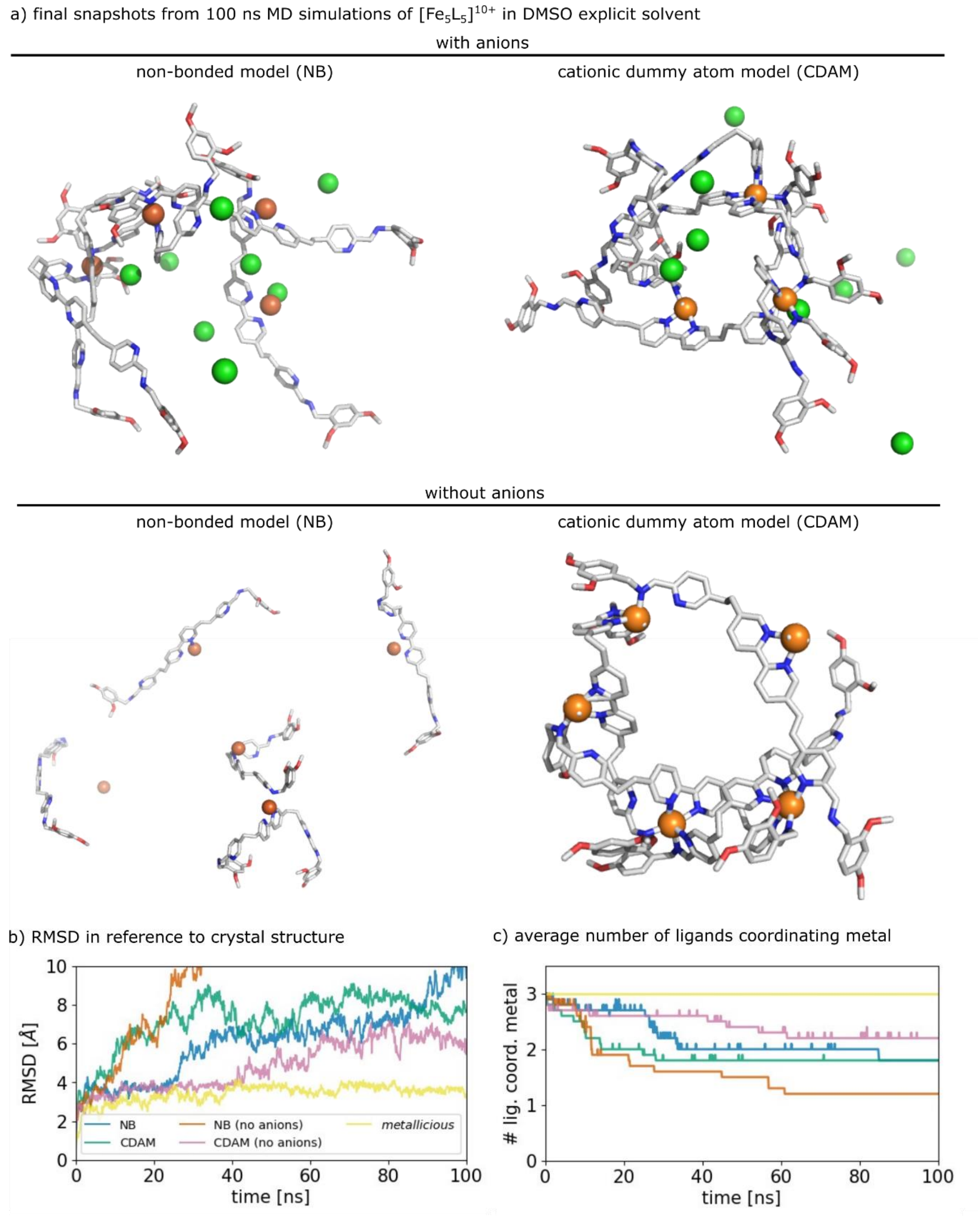

Figure S14. MD simulations of $[Fe_5L_5]^{10+}$ knot in explicit DMSO. (a) Final snapshots for non-bonded (NB) model and cationic dummy atom model (CDAM) of metals with and without chloride (green balls), solvent not shown for clarity. (b) RMSD relative to starting structure for NB model (blue), CDAM (green), NB model without anions (orange), CDAM without anions (purple) and *metallicious* (yellow). (c) Average number of ligands coordinating metals.

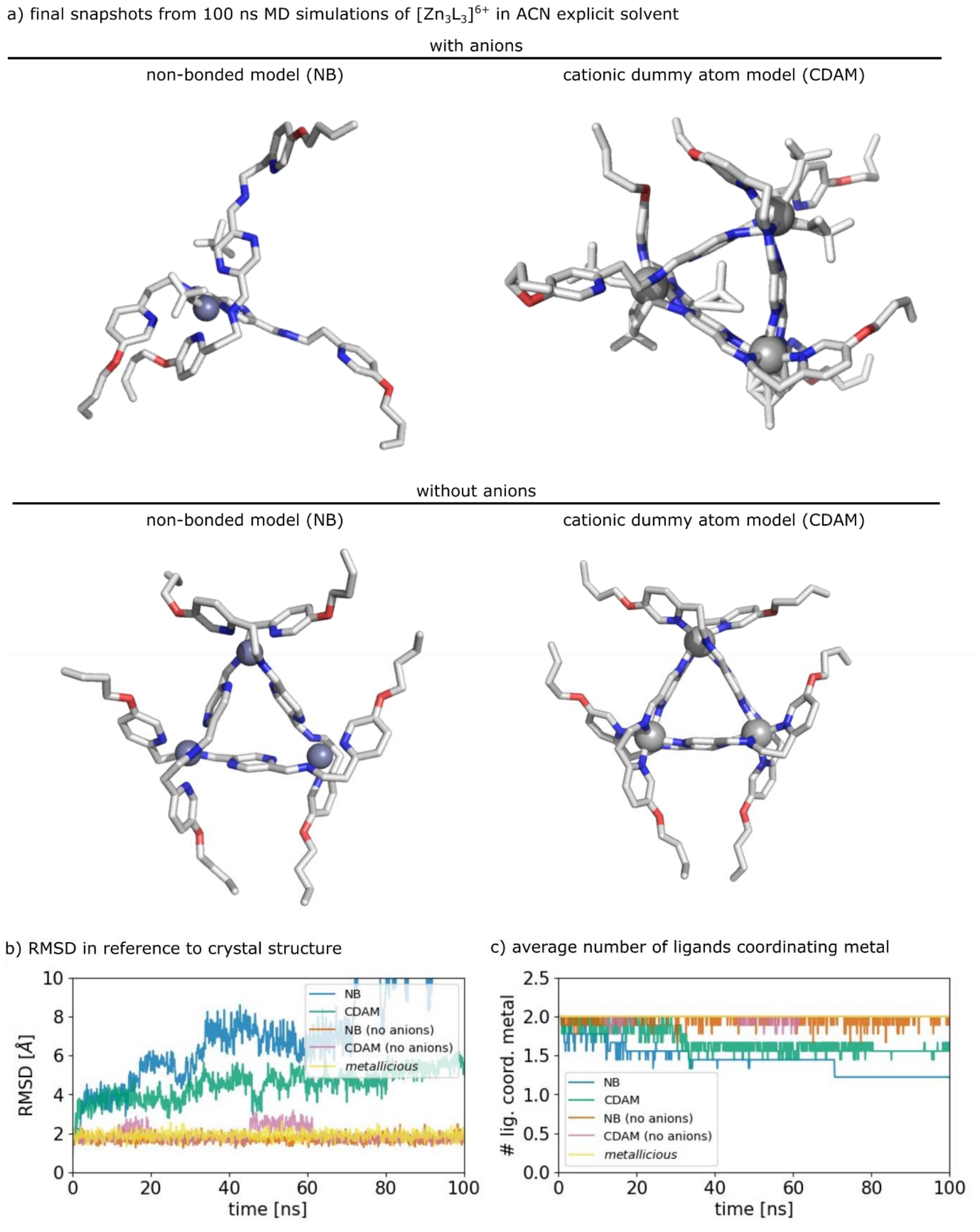

Figure S15. MD simulations of $[Zn_3L_6]^{6+}$ knot in explicit MeCN. (a) Final snapshots for non-bonded (NB) model and cationic dummy atom model (CDAM) of metals with and without BF_4^- (solvent not shown for clarity). (b) RMSD relative to starting structure for NB model (blue), CDAM (green), NB model without anions (orange), CDAM without anions (purple) and *metallicious* (yellow). (c) Average number of ligands coordinating metals.

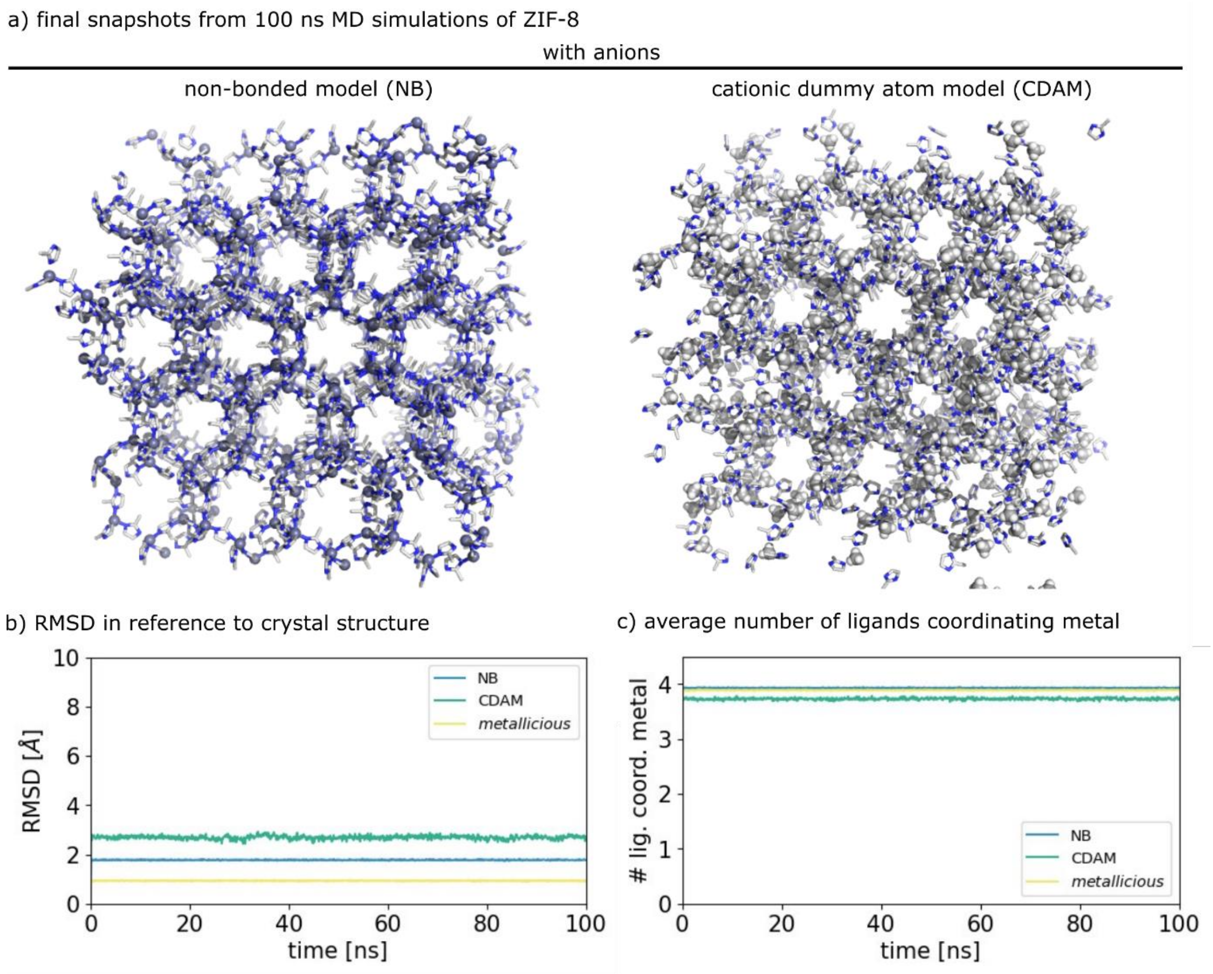

Figure S16. MD simulations of ZIF-8 MOF. (a) Final snapshots for non-bonded (NB) model and cationic dummy atom model (CDAM) of metals. (b) RMSD relative to starting structure for NB model (blue), CDAM (green), and *metallicious* (yellow). (c) Average number of ligands coordinating metals.

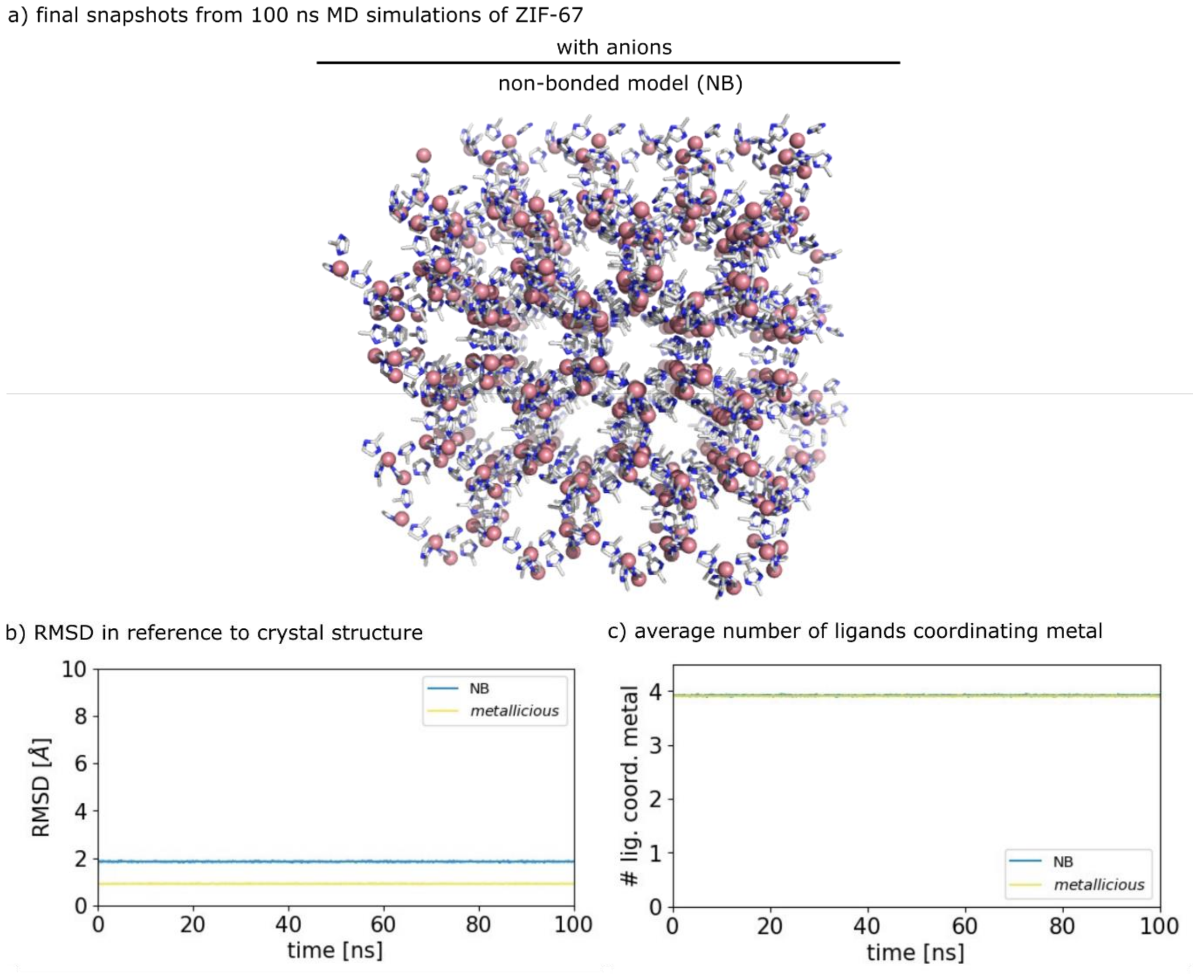

Figure S17. MD simulations of ZIF-67 MOF. (a) Final snapshots for non-bonded. (b) RMSD relative to starting structure for non-bonded (NB) model (blue) and *metallicious* (yellow). (c) Average number of ligands coordinating metals.

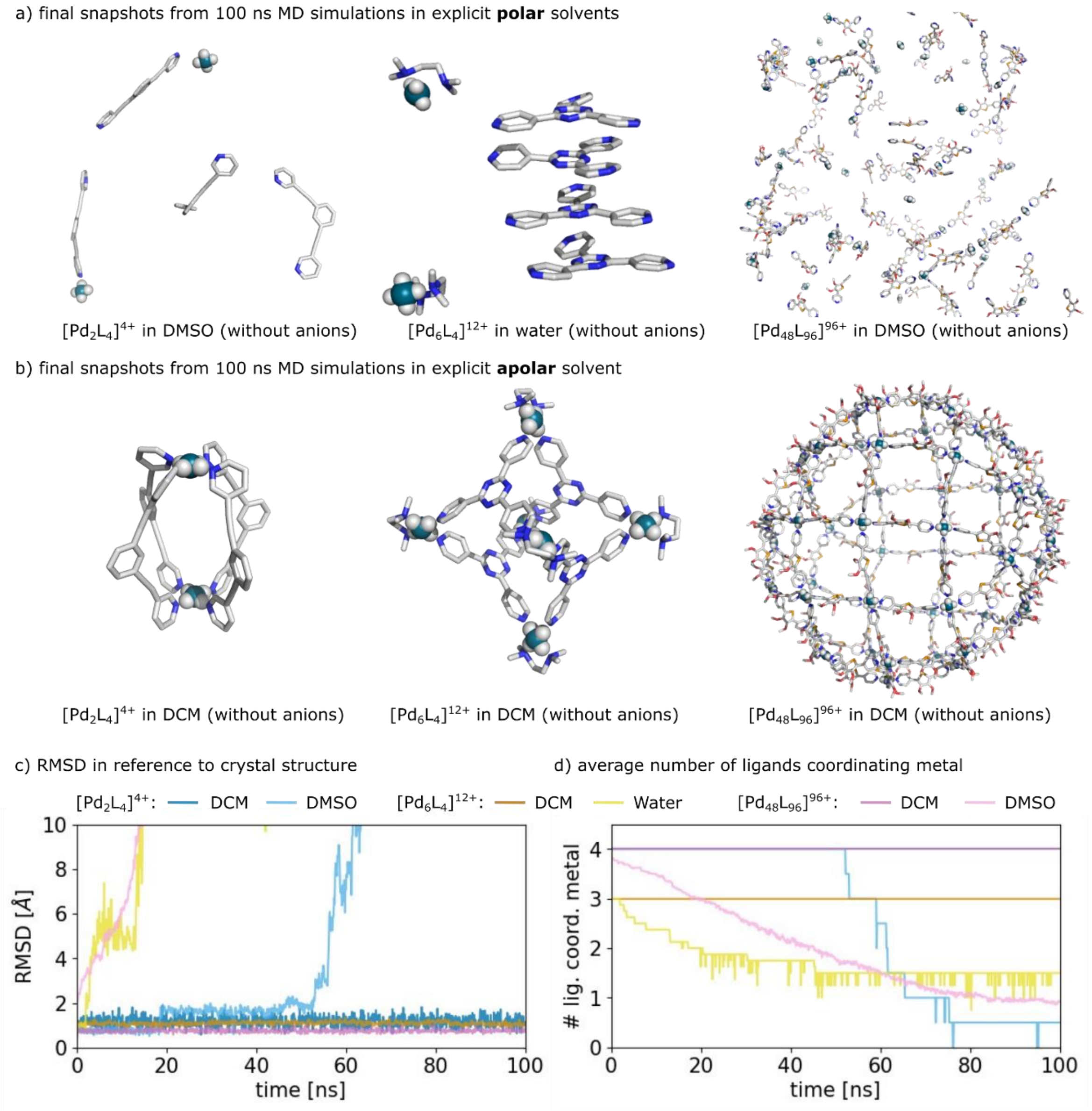

Figure S18. (a-b) Final snapshots from 100 ns MD simulation using cationic dummy model, without anions, of $[Pd_2L_4]^{4+}$ $[Pd_6L_4]^{12+}$ and $[Pd_{48}L_{96}]^{96+}$ in explicit (a) polar solvent (DMSO and water) and (b) apolar solvent (DCM). (c) RMSD relative to starting structure for $[Pd_2L_4]^{4+}$ in DCM (dark blue), DMSO (light blue), $[Pd_6L_4]^{12+}$ in DCM (yellow) and water (orange), $[Pd_{48}L_{96}]^{96+}$ in DCM (purple) and DMSO (pink). (d) Average number of ligands coordinating to the metals.

S4.2.2 Cavity volume

To determine the flexibility of the cages, we have measured relative cavity volumes to average (**Figure S19)**, which were calculated using:

$$V = \frac{V - V_{average}}{V_{average}}, \text{ (Eq. S2)}$$

Where V is the volume of the cavity along the trajectory, and $V_{average}$ is its average value. Cavity volumes were calculated using *C3*.[31] The largest variation was observed for $[Ga_4L_6]^{12-}$, which cavity volume can decrease by 100%, that is to empty cage, and increase by 100%, that is double the cavity size.

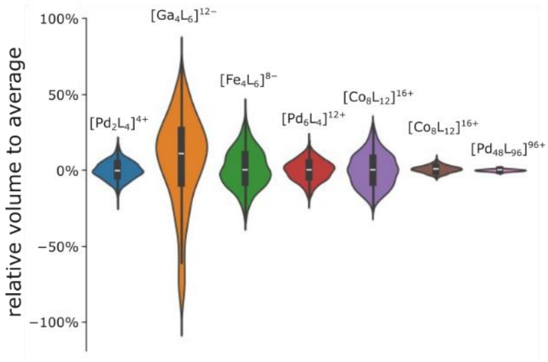

Figure S19. Relative cavity volumes to average were calculated based on Eq. S2. The cavity volumes were calculated using *C3*.[31]

S4.2.3 Radial distribution functions (RDF)

To determine host-guest interactions of counterions with cages and knots, we analyzed radial distribution functions (RDFs) between counterions' centers of mass and metals (see **Table S2** for list of counterions/cages pairs; **Figure S20**); studied MOFs were not analyzed as they did not include ions. The computed RDFs show prominent peaks for $[Pd_2L_4]^{4+}$, $[Zn_3L_3]^{6+}$, and $[Fe_5L_5]^{10+}$, suggesting binding of an anion to the supramolecular structure (**Figure S20)**. $[Pd_6L_4]^{12+}$, $[Co_8L_{12}]^{16+}$, $[Pd_6Ru_8L_{24}]^{28+}$ and $[Pd_{48}L_{96}]^{96+}$ showed less well-defined RDFs peaks, suggesting a less structured arrangement between the cage and anions. The two anionic cages $[Ga_4L_6]^{12-}$ and $[Fe_4L_6]^{4-}$ showed no structure in the RDF plot, suggesting that cations do not interact with the cages.

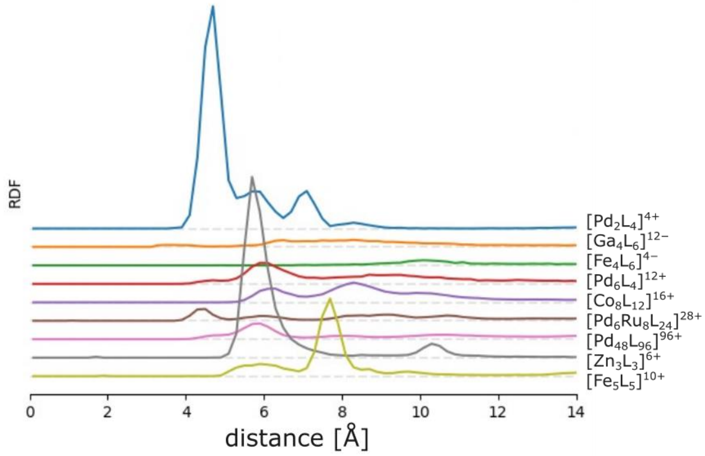

Figure S20. RDFs were calculated for metal centres and counterions from the MD trajectories of all tested supramolecular structures.

REFERENCES

(1) Hagberg, A. A.; Schult, D. A.; Swart, P. J. Exploring Network Structure, Dynamics, and Function Using NetworkX. In *Proceedings of the 7th Python in Science Conference*; Varoquaux, G., Vaught, T., Millman, J., Eds.; Pasadena, CA USA, 2008; pp 11–15.

(2) Bayly, C. I.; Cieplak, P.; Cornell, W. D.; Kollman, P. A. A Well-Behaved Electrostatic Potential Based Method Using Charge Restraints for Deriving Atomic Charges: The RESP Model. *J. Phys. Chem.* **1993**, *97* (40), 10269–10280. https://doi.org/10.1021/j100142a004.

(3) August, D. P.; Nichol, G. S.; Lusby, P. J. Maximizing Coordination Capsule–Guest Polar Interactions in Apolar Solvents Reveals Significant Binding. *Angew. Chem. Int. Ed.* **2016**, *55* (48), 15022–15026. https://doi.org/10.1002/anie.201608229.

(4) Pluth, M. D.; Johnson, D. W.; Szigethy, G.; Davis, A. V.; Teat, S. J.; Oliver, A. G.; Bergman, R. G.; Raymond, K. N. Structural Consequences of Anionic Host-Cationic Guest Interactions in a Supramolecular Assembly. *Inorg. Chem.* **2009**, *48* (1), 111–120. https://doi.org/10.1021/ic8012848.

(5) Mal, P.; Breiner, B.; Rissanen, K.; Nitschke, J. R. White Phosphorus Is Air-Stable within a Self-Assembled Tetrahedral Capsule. *Science* **2009**, *324* (5935), 1697–1699. https://doi.org/10.1126/science.1175313.

(6) Takezawa, H.; Murase, T.; Resnati, G.; Metrangolo, P.; Fujita, M. Halogen-Bond-Assisted Guest Inclusion in a Synthetic Cavity ** Angewandte. **2015**, *1* (3), 8411–8414. https://doi.org/10.1002/anie.201500994.

(7) Metherell, A. J.; Cullen, W.; Williams, N. H.; Ward, M. D. Binding of Hydrophobic Guests in a Coordination Cage Cavity Is Driven by Liberation of " High-Energy " Water. **2018**, 1554–1560. https://doi.org/10.1002/chem.201704163.

(8) Wu, K.; Li, K.; Hou, Y. J.; Pan, M.; Zhang, L. Y.; Chen, L.; Su, C. Y. Homochiral D4-Symmetric Metal–Organic Cages from Stereogenic Ru(II) Metalloligands for Effective Enantioseparation of Atropisomeric Molecules. *Nat. Commun.* **2016**, *7* (1), 10487. https://doi.org/10.1038/ncomms10487.

(9) Fujita, D.; Ueda, Y.; Sato, S.; Mizuno, N.; Kumasaka, T.; Fujita, M. Self-Assembly of Tetravalent Goldberg Polyhedra from 144 Small Components. *Nature* **2016**, *540* (7634), 563–566. https://doi.org/10.1038/nature20771.

(10) Ayme, J. F.; Beves, J. E.; Leigh, D. A.; McBurney, R. T.; Rissanen, K.; Schultz, D. Pentameric Circular Iron(II) Double Helicates and a Molecular Pentafoil Knot. *J. Am. Chem. Soc.* **2012**, *134* (22), 9488–9497. https://doi.org/10.1021/ja303355v.

(11) Zhang, L.; August, D. P.; Zhong, J.; Whitehead, G. F. S.; Vitorica-Yrezabal, I. J.; Leigh, D. A. Molecular Trefoil Knot from a Trimeric Circular Helicate. *J. Am. Chem. Soc.* **2018**, *140* (15), 4982–4985. https://doi.org/10.1021/jacs.8b00738.

(12) Morris, W.; Stevens, C. J.; Taylor, R. E.; Dybowski, C.; Yaghi, O. M.; Garcia-Garibay, M. A. NMR and X-Ray Study Revealing the Rigidity of Zeolitic Imidazolate Frameworks. *J. Phys. Chem. C* **2012**, *116* (24), 13307–13312. https://doi.org/10.1021/jp303907p.

(13) Banerjee, R.; Phan, A.; Wang, B.; Knobler, C.; Furukawa, H.; O'Keeffe, M.; Yaghi, O. M. High-Throughput Synthesis of Zeolitic Imidazolate Frameworks and Application to CO2 Capture. *Science* **2008**, *319*, 939–943. https://doi.org/10.1126/science.1152516.

(14) Abraham, M. J.; Murtola, T.; Schulz, R.; Pall, S.; Smith, J. C.; Hess, B.; Lindah, E. Gromacs: High Performance Molecular Simulations through Multi-Level Parallelism from Laptops to Supercomputers. *SoftwareX* **2015**, *1–2*, 19–25. https://doi.org/10.1016/j.softx.2015.06.001.

(15) Allen, A. E. A.; Payne, M. C.; Cole, D. J. Harmonic Force Constants for Molecular Mechanics Force Fields via Hessian Matrix Projection. *J. Chem. Theory Comput.* **2018**, *14* (1), 274–281. https://doi.org/10.1021/acs.jctc.7b00785.

(16) Onufriev, A.; Bashford, D.; Case, D. A. Exploring Protein Native States and Large-Scale Conformational Changes with a Modified Generalized Born Model. *Proteins Struct. Funct. Genet.* **2004**, *55* (2), 383–394. https://doi.org/10.1002/prot.20033.

(17) Van Der Spoel, D.; Lindahl, E.; Hess, B.; Groenhof, G.; Mark, A. E.; Berendsen, H. J. C. GROMACS: Fast, Flexible, and Free. *J. Comput. Chem.* **2005**, *26* (16), 1701–1718. https://doi.org/10.1002/jcc.20291.

(18) Izadi, S.; Anandakrishnan, R.; Onufriev, A. V. Building Water Models : A Different Approach. *J. Phys. Chem. Lett.* **2014**, *5*, 3853–3871.

(19) van der Spoel, D.; van Maaren, P. J.; Caleman, C. GROMACS Molecule & Liquid Database. *Bioinformatics* **2012**, *28* (5), 752–753. https://doi.org/10.1093/bioinformatics/bts020.

(20) Wang, J.; Wang, W.; Kollman, P. A.; Case, D. A. Automatic Atom Type and Bond Type Perception in Molecular Mechanical Calculations. *J. Mol. Graph. Model.* **2006**, *25* (2), 247–260. https://doi.org/10.1016/j.jmgm.2005.12.005.

(21) Bussi, G.; Donadio, D.; Parrinello, M. Canonical Sampling through Velocity Rescaling. *J. Chem. Phys.* **2007**, *126* (2007), 014101. https://doi.org/10.1063/1.2408420.

(22) Bernetti, M.; Bussi, G. Pressure Control Using Stochastic Cell Rescaling. *J. Chem. Phys.* **2020**, *153* (11), 114107. https://doi.org/10.1063/5.0020514.

(23) Darden, T.; York, D.; Pedersen, L. Particle Mesh Ewald: An N -Log(N) Method for Ewald Sums in Large Systems. *J. Chern. Phys.* **1993**, *98* (12), 10089–10092.

(24) Essmann, U.; Perera, L.; Berkowitz, M. L.; Darden, T.; Lee, H.; Pedersen, L. G. A Smooth Particle Mesh Ewald Method. *J. Chem. Phys.* **1995**, *103* (19), 8577–8593. https://doi.org/10.1063/1.470117.

(25) Michaud-Agrawal, N.; Denning, E. J.; Woolf, T. B.; Beckstein, O. MDAnalysis: A Toolkit for the Analysis of Molecular Dynamics Simulations. *J. Comput. Chem.* **2011**, *32* (10), 2319–2327. https://doi.org/10.1002/jcc.

(26) Gowers, R. J.; Linke, M.; Barnoud, J.; Reddy, T. J. E.; Melo, M. N.; Seyler, S. L.; Domański, J.; Dotson, D. L.; Buchoux, S.; Kenney, I. M.; et al. MDAnalysis: A Python Package for the Rapid Analysis of Molecular Dynamics Simulations. In *Proceedings of the 15th Python in Science Conference*; Benthall, S., Rostrup, S., Eds.; 2016; pp 98–105. https://doi.org/10.25080/Majora-629e541a-00e.

(27) Yoneya, M.; Yamaguchi, T.; Sato, S.; Fujita, M. Simulation of Metal-Ligand Self-Assembly into Spherical Complex M6L8. *J. Am. Chem. Soc.* **2012**, *134* (35), 14401–14407. https://doi.org/10.1021/ja303542r.

(28) Yoneya, M.; Tsuzuki, S.; Yamaguchi, T.; Sato, S.; Fujita, M. Coordination-Directed Self-Assembly of M12L24 Nanocage: Effects of Kinetic Trapping on the Assembly Process. *ACS Nano* **2014**, *8* (2), 1290–1296. https://doi.org/10.1021/nn404595j.

(29) Tironi, I. G.; Sperb, R.; Smith, P. E.; Van Gunsteren, W. F. A Generalized Reaction Field Method for Molecular Dynamics Simulations. *J. Chem. Phys.* **1995**, *102* (13), 5451–5459. https://doi.org/10.1063/1.469273.

(30) Krokidas, P.; Castier, M.; Moncho, S.; Brothers, E.; Economou, I. G. Molecular Simulation Studies of the Diffusion of Methane, Ethane, Propane, and Propylene in ZIF-8. *J. Phys. Chem. C* **2015**, *119* (48), 27028–27037. https://doi.org/10.1021/acs.jpcc.5b08554.

(31) Martí-Centelles, V.; Piskorz, T. K.; Duarte, F. *CageCavityCalc (C3): A Computational Tool for Calculating and Visualizing Cavities in Molecular Cages.*; 2024. https://doi.org/10.26434/chemrxiv-2024-fmlx0.
